# Determining subpopulation methylation profiles from bisulfite sequencing data of heterogeneous samples using DXM

**DOI:** 10.1093/nar/gkab516

**Published:** 2021-06-22

**Authors:** Jerry Fong, Jacob R Gardner, Jared M Andrews, Amanda F Cashen, Jacqueline E Payton, Kilian Q Weinberger, John R Edwards

**Affiliations:** Center for Pharmacogenomics, Department of Medicine, Washington University School of Medicine, St. Louis, MO, USA; Center for Data Science for Improved Decision Making, Department of Computer Science, Cornell University, Ithaca, NY, USA; Department of Pathology and Immunology, Washington University School of Medicine, St. Louis, MO, USA; Oncology Division, Department of Medicine, Washington University School of Medicine, St. Louis, MO, USA; Department of Pathology and Immunology, Washington University School of Medicine, St. Louis, MO, USA; Center for Data Science for Improved Decision Making, Department of Computer Science, Cornell University, Ithaca, NY, USA; Center for Pharmacogenomics, Department of Medicine, Washington University School of Medicine, St. Louis, MO, USA

## Abstract

Epigenetic changes, such as aberrant DNA methylation, contribute to cancer clonal expansion and disease progression. However, identifying subpopulation-level changes in a heterogeneous sample remains challenging. Thus, we have developed a computational approach, DXM, to deconvolve the methylation profiles of major allelic subpopulations from the bisulfite sequencing data of a heterogeneous sample. DXM does not require prior knowledge of the number of subpopulations or types of cells to expect. We benchmark DXM’s performance and demonstrate improvement over existing methods. We further experimentally validate DXM predicted allelic subpopulation-methylation profiles in four Diffuse Large B-Cell Lymphomas (DLBCLs). Lastly, as proof-of-concept, we apply DXM to a cohort of 31 DLBCLs and relate allelic subpopulation methylation profiles to relapse. We thus demonstrate that DXM can robustly find allelic subpopulation methylation profiles that may contribute to disease progression using bisulfite sequencing data of any heterogeneous sample.

## INTRODUCTION

DNA methylation changes have been implicated in a variety of human diseases, including cancer (1). Methylation measurements are typically made from heterogenous samples consisting of multiple cell-types, which each have unique methylation patterns. As such, it is frequently difficult to interpret whether an observed change in methylation is due to a shift in sample composition or due to a true change in the methylation state of an underlying cell-type. For example, tumors are comprised of both normal cell types and cancer subclones. These subclones can acquire changes that increase their fitness, leading to faster cancer progression, treatment resistance and worse patient prognosis (2,3). Though cancer subclones have generally been described with respect to genetic alterations, in principle epigenetic alterations such as DNA methylation could alter the expression of key genes in a subclone and impact its fitness. Moreover, in chronic lymphocytic leukemia (CLL), diffuse large B-cell lymphoma (DLBCL), acute myeloid leukemia (AML), Ewing Sarcoma, and glioblastoma, clonal heterogeneity in DNA methylation is associated with worse patient outcome (4–8). Unfortunately, even though subclonal methylation changes may be expected to underlie this observed heterogeneity, current methods do not effectively analyze subclonal methylation patterns from the bisulfite sequencing data of heterogeneous samples. One approach to address sample heterogeneity is fluorescence-activated cell-sorting (FACS), but it is dependent on surface markers to distinguish cell types, which may not be known *a priori* or even exist for many cancer subclones. Hypothetically, single-cell bisulfite sequencing can address sample heterogeneity (9), but it is technically challenging and expensive. While this technology continues to develop, there remain hundreds of bisulfite sequencing datasets that have already been generated and are being generated that would remain useful to study. To understand the impact of methylation changes measured in these samples we thus need a computational approach to identify the number of underlying allelic subpopulations and their respective methylation profiles from the methylation data of a heterogeneous input sample.

Though extensive methods have been developed to describe subclonal architecture with respect to mutations or copy number variants (CNVs) (for a review see (10)), these methods cannot be easily adapted to methylation analysis since they typically assume subclonal events occur independently and are relatively rare. Unlike mutations or CNVs, methylation changes often occur in blocks of multiple CpGs over a short range called differentially methylated regions (DMRs) (11,12). Additionally, there are many more aberrant changes in methylation than mutations or CNVs, with frequently >100 000 DMRs observed in solid tumors as compared to at most ∼10 000 mutations or ∼100 CNVs (13–15). Thus we need approaches that specifically model subclonal DNA methylation data.

Several analysis methods to determine the cellular composition and methylation profiles of subpopulation level events have been developed for epigenome-wide association studies (EWAS) (16–20). However, EWAS uses array-based technologies that probe the methylation state of 3% of the CpGs in the human genome, and as such, these approaches use assumptions and error-models that are not appropriate for sequence data. For example, they do not consider the strong local correlations of methylation changes, since adjacent probes are frequently >1 kb apart (21). Consequently, there are not good options for those interested in deconvolving sequencing-based DNA methylation data such as obtained through WGBS, RRBS or capture methods.

**Figure 1.**
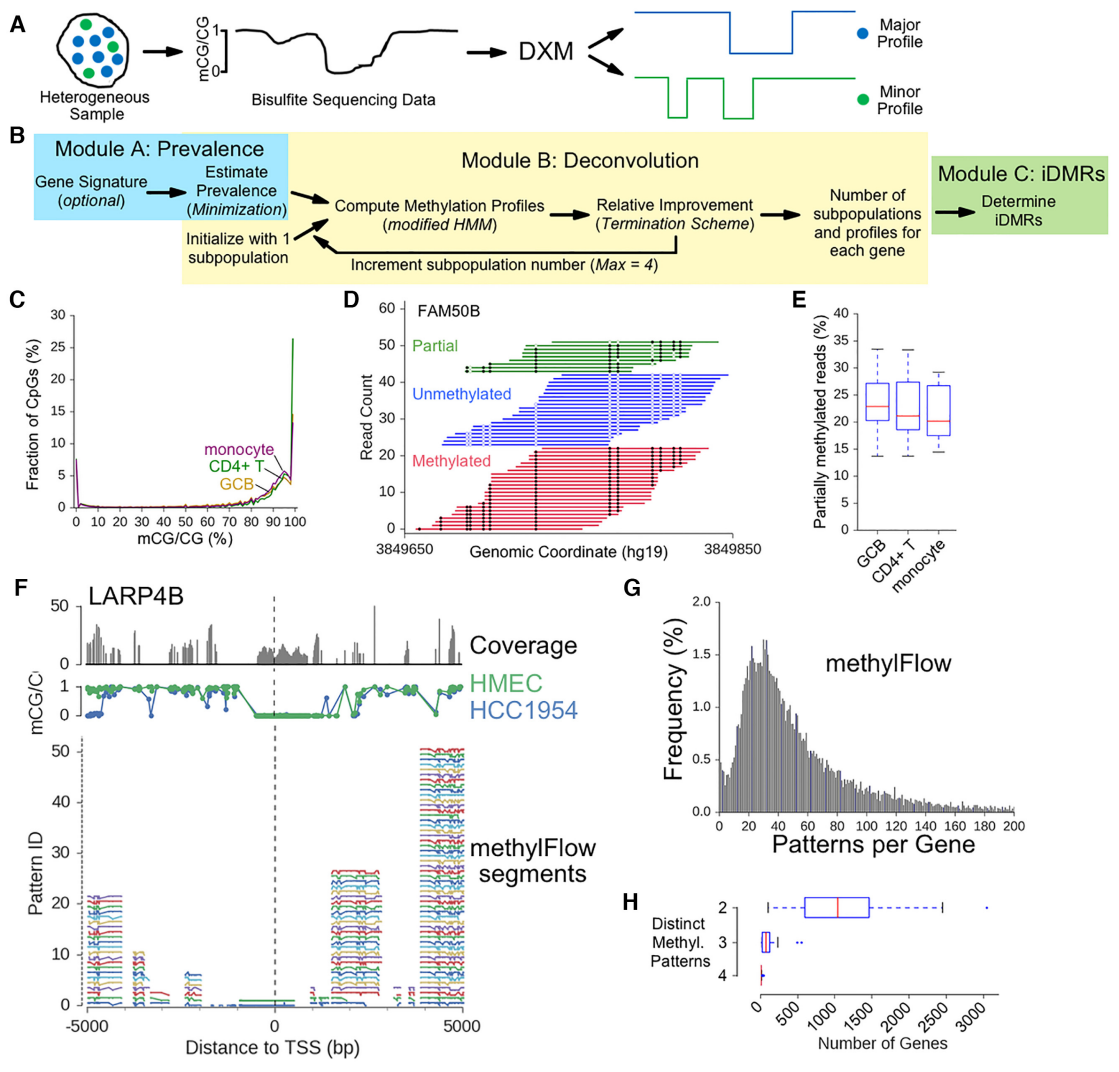
DXM scheme and design considerations. (**A**) DXM takes bisulfite sequencing data from a heterogeneous sample and identifies its underlying allelic subpopulation methylation profiles and the number of subpopulations. (**B**) DXM consists of three modules: Module A estimates the prevalence, Module B solves subpopulation methylation profiles, and Module C calls intrasample DMRs (i-DMRs). (**C**) Fractional methylation distribution for sorted cell types. 12.4%, 15.5% and 22.7% of CpGs are partially methylated (i.e. the fraction of CpGs between 20–80% methylation) in CD4+ T-cells (green), GCB cells (gold), and monocytes (purple), respectively. (**D**) Bisulfite sequencing reads across part of the imprinting control region for FAM50B in germinal center B-cells (GCBs) from BEP. Open circles are unmethylated CpGs and closed circles are methylated. (**E**) The percentage of partially methylated reads at 19 well-characterized imprinted loci in different sorted cell types from BEP. (**F**) methylFlow (MF) outputs for LARP4B from analysis of a 22× coverage HMEC-HCC1954 mixture (35:65). For individual segments, MF outputs between 1 and 52 potential profiles. (**G**) The most profiles in any individual segment by MF for all promoters in a 22× coverage HMEC-HCC1954 mixture (35:65). (**H**) Number of gene promoters with DMRs identified between or among different sorted cell types from BEP (CD4T, CD8T, erythroblast, eosinophil, hematopoietic multiprogenitor, GCB, megakaryocyte, monocyte, osteoclast). Promoters were considered to have distinct methylation patterns if there was a DMR identified between each pair of cells considered (e.g. for cell types A, B and C, there are three distinct patterns if there is a DMR between A–B, B–C and A–C). 17 450 total genes were considered.

Thus, we have developed DXM (**D**econvolution of Subpopulations E**x**isting in **M**ethylation Data), a novel deconvolution strategy to identify the major allelic subpopulations and their respective methylation profiles from a heterogeneous sample (Figure 1A and B). DXM does not require explicit prior knowledge of the number of subpopulations or what types of cells to expect, and it provides a framework for considering methylation differences across multiple CpGs at the single-CpG resolution offered by bisulfite sequencing data. We benchmarked DXM on a wide set of simulated mixtures using bisulfite sequencing reads from sorted hematopoietic cell types and found that DXM outperformed methylPurify (22), another method developed for subclonal analysis of bisulfite sequencing data. We demonstrate that DXM can be used to study allele-specific methylation in the contexts of X-inactivation and imprinting. We further conducted Agilent Methyl-Seq bisulfite sequencing analysis in four samples from patients with DLBCL, a B-cell lymphoma derived from germinal center B cells. We validated that DXM predictions for subpopulation methylation profiles were recapitulated in relevant sorted CD4^+^ T and CD19^+^ B cells from these samples. As proof-of-concept, we applied DXM to bisulfite sequencing data from a cohort of 31 DLBCL samples (5) to highlight how DXM can be used to analyze subpopulation methylation in heterogeneous cancer samples and relate them to relapse.

## MATERIALS AND METHODS

### Public datasets

Bisulfite sequencing data was downloaded from the Roadmap Epigenomics project (23) (REP), the Blueprint Epigenome Project (24) (BEP), ENCODE (25) and GEO (Accession: GSE29069 (26), GSE66329 (27), GSE75868 (for the WIBR3 cell-lines) (28). A full list is provided in Supplementary File 1 (Supplementary_File_1.xlsx).

### DXM

Using sequencing-based DNA methylation data from heterogeneous samples as input, DXM deconvolves data across a set of user-defined regions and outputs the number of allelic subpopulations as well as the methylation profile for each subpopulation across each region (Figure 1A and B). We adopt an iterative scheme to solve for the number of subpopulations, beginning with only 1 major subpopulation, and adding additional subpopulations provided that they do not cause an overfit. This approach does not assume how many underlying subpopulations are present *a priori*, but it takes an Occam's Razor approach in determining the minimal number of subpopulations that can reasonably explain the observed data.

#### DXM input

DXM uses processed data as input, consisting of a BED-like format containing tab-delimited columns of chromosome names, start position, end position, methylation level (mCG/CG), sequencing coverage, and region id. DXM performs deconvolution over user-specified intervals (e.g. promoter regions, CGIs, or enhancers) and is compatible with any reference genome. While input data does not need to be filtered for a specific coverage cutoff, we recommend a coverage of at least 4 reads per CpG for more reliable results. Typically, we recommend collapsing CpG data across strands to increase reliability of the methylation estimates, but this is not required. Additional pre-processing details for specific experiments are detailed in their respective sections below.

#### Minimization

We next estimate the best possible prevalence of subpopulations from the distribution of all fractional methylation values detected. We minimize the L1-difference between the expected underlying fractional methylation values and the original methylation distribution. The expected underlying fractional methylation values represent a set of all possible combinations for detected methylation given that each subpopulation will be methylated or not. For example, if there are two subpopulations with prevalence of 0.3 or 0.7 in the sample, then the expected fractional methylation values detected are {0, 0.3, 0.7, 1}. In the (unlikely) event of a tie, the solution with the smallest possible subpopulation is selected. After minimization, each region is solved independently for its methylation profiles.

#### Modified hidden markov model

Given the number of underlying subpopulations and their expected prevalence, we solve for the most likely methylation profiles by applying the Viterbi algorithm to a modified Hidden Markov Model (HMM). An HMM is well-suited to model sequence data with local correlations, as exhibited by DNA methylation (Supplementary Figure S1a–d) (29). In brief, the HMM in DXM first considers how likely a given methylation profile would contribute to the observed bisulfite sequencing data. It then extends this logic for how likely a mixture of these methylation profiles would contribute to the observed data. The underlying methylation sequence of the CpGs of all profiles represents the state-sequence. The transition probabilities are the likelihood that a cell type would have a certain underlying methylation sequence for its CpGs, given the probability that two nearby CpG sites have the same methylation state as a function of the distance between them. The 1 are the likelihood of a CpG’s underlying methylation state across all subpopulations contributing to the observed bisulfite sequencing data as modeled by a beta-binomial distribution. This setup allows the HMM to holistically consider methylation data from all CpGs in the region and account for data quality of each CpG (coverage). The HMM states, transitions, and emissions are described in greater detail below.

#### HMM states

For our modified HMM, if there are *d* subpopulations to consider, a state is a length-*d* vector of the binary methylation values of each underlying subpopulation at a given CpG.

#### HMM transition probabilities

The transition probability represents the change in methylation of each underlying subpopulation from one CpG to an adjacent CpG. To calculate the transition probability, we have defined a set of 2 × 2 transition matrices that capture transitions of one subpopulation's methylation state from one CpG to the next. From this set, the transition matrix is selected based on the distance between CpGs, which captures correlations in methylation and distance seen in Supplementary Figure S1a–d. We consider each subpopulation's transition to be independent and identically distributed, so the transition probability is ultimately calculated from *d* identical transition matrices.

To ensure robustness, transition probabilities were trained on somatic tissue and cell-line datasets available via REP, and all testing was conducted on datasets of different cell types available for BEP (Supplementary_File_1.xlsx) or GSE29069. Briefly, for all CpGs in a dataset, the detected fractional methylation was rounded to the closest binary methylation state (fully unmethylated or fully methylated). Transitions between binary methylation states of adjacent CpGs were then counted (empirical sampling). We elected to group all transitions greater than 1000 bp in the same category when selecting transition probabilities to use. Notably, this training includes correlations across genomic elements, including open-sea regions and CpG islands, as we did not observe a large difference in correlations across genic or intergenic regions (Supplementary Figure S1b–d). We do not utilize any cell type-specific transition probabilities for underlying subpopulations. Importantly, we observed similar correlations across multiple datasets, including different assays for methylation sequencing (WGBS, RRBS, Agilent Methyl-Seq), suggesting DXM can be applied to any genome-wide or reduced representation methylation sequencing dataset.

(1) $$\begin{eqnarray*}P\left( {b,n} \right) &=& \mathop \sum \limits_{i = 0}^n \mathop \sum \limits_{j = 0}^{{\rm{min}}\left( {i,b} \right)}\binom{n}{i}{p^i}{\left( {1 - p} \right)^{n - i}}\nonumber\\ &&\quad*\frac{\binom{i}{j}\binom{n-i}{b-j}}{\binom{n}{b}}*m(i,j)*u(n-i,b-j)\end{eqnarray*}$$

#### Emission probabilities

The emission probabilities, *P*(*b,n*), at each CpG site were calculated from Equation (1), which has terms to capture the prevalence of each subpopulation, the binomial sampling error, and measurement errors. In this equation, *n* is the total number of reads (coverage) at that CpG site, *b* is the number of methylated reads, *i* is the number of reads that came from the underlying methylated state, *m*(*i*, *j*) is the probability that *j* of these *i* reads are correctly measured as methylated reads, *u*(*n* – *i*, *b* – *j*) is the probability that *b* – *j* of the *n* – i unmethylated reads are incorrectly measured from the unmethylated state (i.e. measured as methylated), and *p* is the prevalence of all subpopulations with an underlying methylated state. Assuming reads are independently and identically distributed among allelic subpopulations as a function of prevalence, reads can be grouped by whether they came from an allelic subpopulation whose underlying state was methylated or not. Thus, if *i* reads came from subpopulations with an underlying state of methylated, *n – i* come from those with an underlying state as unmethylated, and this case follows a binomial distribution, where *p* is the prevalence of all subpopulations with an underlying methylated state. Of these *i* reads, *j* may be observed as methylated, and assuming all reads are independent, the probability of this event follows a hypergeometric distribution. Lastly, the probability that *j* of *i* reads are observed as methylated, given that the expected underlying state is fully methylated, is modeled with a beta-binomial distribution *m*(*i*, *j*). A separate beta-binomial distribution, *u*(*n* – *i*, *b* – *j*), models a similar probability that *b – j* of *n* – *i* reads are observed as methylated given an expected underlying state of fully unmethylated. Determination of the beta-priors for these beta-binomial distributions is provided below (see below in *Beta-binomial fit*). Summing across all valid combinations of *i, j* gives the final emission probabilities. We use log-probabilities in all calculations to ensure precision.

#### Termination scheme

DXM determines the minimum number of subpopulations that explain the data by terminating when adding another allelic subpopulation no longer improves the fit to the data. Let $p( x )$ represent the probability that the state-sequence *x* correctly explains the data and let *x_r_* represent the most likely state-sequence for *r* subpopulations. DXM will consider an additional subpopulation to be valid if there is an increase in likelihood, that is if $p( {x = {x_{r + 1}}} ) >p( {x = {x_r}} )$. By Bayes’ rule, this can be written as $p( {x = {x_{r + 1}} \mid r + 1} )*p( {r + 1} ) >p( {x = {x_r} \mid r} )*p( r )$, where $p( r )$ is the probability that *r* subpopulations best explain the underlying data. In the uninformed case, we would have a uniform prior on the number of subpopulations to expect, e.g. $p( r ) = p( {r + 1} )$, and thus, the comparison is $p( {x = {x_{r + 1}} \mid r + 1} ) >p( {x = {x_r} \mid r} )$. The conditional probability of a state-sequence *x_r_* explaining the data given *r* subpopulations, $p( {x = {x_r} \mid r} ),$ is solved by the Viterbi algorithm, so termination is achieved when the relative probability of a solution is worse. Though it is possible that our scheme does not yield the global solution {e.g. $p( {x = {x_{r + 1}} \mid r + 1} ) \le p( {x = {x_r} \mid r} )$ but $p( {x = {x_{r + 2}} \mid r + 2} ) >p( {x = {x_r} \mid r} )$}, we have not observed this in practice and believe the case to be highly unlikely for reasonably well-behaved beta-binomial distributions. Additionally, this scheme is guaranteed to converge. Consider the case where an additional subpopulation has the same methylation profile as an existing one. In this case, the emission probability would remain unchanged, but the transition probabilities would be less likely since there is an additional subpopulation to consider. Since there are a finite number of possible methylation profiles for *g* CpGs (2^g^), this case is guaranteed, though in practice we need to consider far fewer than 2^*g*^ methylation profiles (e.g. four total profiles).

#### DXM output

The output of DXM includes an estimate to the number of underlying subpopulations, their respective methylation profiles across each user-defined region in BED-like format, and an estimate of their relative prevalence in the sample. Given the utility of identifying differentially methylated regions (DMRs), we have also provided a post-hoc utility to identify intrasample DMRs between or among identified subpopulation methylation profiles (i-DMRs). Throughout this work we require i-DMRs to be least 50bp in length, contain at least three CpGs, and have at least 90% of the CpGs are differentially methylated. These are similar to the filtering parameters used by the DMR-caller DSS (30). DXM i-DMRs can be ranked based on their improvement in relative probability when modeled with two subpopulations as compared to 1, given as $p( {x = {x_2} \mid 2\;} ) - p( {x = {x_1} \mid 1} )$, which provides compatibility with DXM results for analyses such as gene set enrichment.

### Beta-Binomial fit

An empirical beta-prior for use in DXM was drawn from training data from REP. This reflects an expectation of how fractional methylation of a given CpG is detected across all reads. In a bisulfite sequencing experiment, we consider that every read represents an independent sampling of the methylation value of the given CpG. For an expected unmethylated state, every CpG with percent methylation between 0% and 40% was considered; similarly, 60–100% were the cutoffs for an expected methylated state. The methylation of each CpG was rounded to the nearest 0.5%, and these discrete empirical distributions were then normalized. To find a suitable beta prior for this distribution, the probability density function of a given beta distribution was evaluated at each domain value in the empirical distribution (e.g. every 0.5% methylation). This new distribution was then normalized, and the L1-difference between it and the empirical distribution was minimized. All alpha/beta parameters for underlying beta distributions were fixed as integers, and a linear combination of beta distributions was employed to better capture spiking behavior near the edges of the distribution (e.g. 0% or 100% methylation) and a small spike around 50% methylation (due to imprinting and allele-specific methylation).

### Region definitions

#### Gene promoter windows

Genes were defined using RefSeq (31) annotations, where only genes (transcripts) with ‘cmpl’ annotations for both the coding start and end sites were used. For genes with multiple transcription start sites (TSS), only the first was considered. Only genes on autosomal chromosomes were considered for analysis. Gene promoter windows were then defined as the region ±5 kb from the annotated TSS. While there is no agreed upon definition of a gene promoter, this region was chosen because it is larger than available definitions and should therefore include the entire promoter for analysis. Additionally, this window has previously been shown to be useful for predicting expression changes from DNA methylation (32).

#### Imprinted regions

Imprinted regions were taken from a list of known imprinted regions (Table 1 in Court *et al.* 2014) (33).

#### X-inactivated CpG islands

CpG islands on chromosome X were defined by UCSC annotations. These were then filtered for association with genes as done for gene promoter windows (refGene.txt file from UCSC with a ‘cmpl’ annotation for coding start and end sites.).

#### Enhancers

For each cell-type, we obtained H3K27Ac and H3Kme1 ChIP-Seq data from BEP (GCB, EGAD00001002442; Monocyte, EGAD00001002523). Active enhancers were defined as regions with overlapping H3K27Ac and H3Kme1 peaks (using bedtools intersect). Enhancers were further annotated as cell-type specific or common for pairs of cell-types based on whether they overlapped (bedtools intersect, using an outer join to consider as wide an overlap as possible). To remove any regions that overlapped promoters, we used bedtools to remove any enhancers that overlapped the region ±1 kb around any TSS (refSeq, genes with a ‘cmpl’ CDS start and end annotation). Finally, only autosomal enhancers with methylation data at ≥4 CpGs with ≥20 reads were used for analysis.

### Methylation-level mixture simulations

First, reference cell-type profiles for HMEC (Human mammary epithelial cells), CD4+ T and HCC1954 breast cancer cells were generated by taking the average methylation level (mCG/CG) for each CpG from published data (see Supplementary_File_1.xlsx for accession numbers) and rounding to 0 or 1 to binarize the profile for each individual sample. CpGs were only considered if there were at least 4 reads. For each simulation at a fixed prevalence and a fixed coverage, 1000 gene promoter regions (±5 kb from the TSS) were randomly selected. The mixed methylation level (mCG/CG) of each CpG was then computed as the dot product of the prevalences and methylation values. Binomial sampling was then used to simulate the measured methylation value of the mixed sample based on the chosen coverage. DXM was then used to deconvolve the mixture.

### Read-level mixture simulations

For simulated mixture generation, bisulfite sequencing reads (from BEP or GSE29069 datasets) were mapped with bsmap2.90 (34) against the hg19 genome using the -R flag. Mapped bisulfite sequencing reads were coordinate-sorted with tabix (35), after which all reads overlapping the TSS ± 10 kb window of genes were taken for input (a larger window was used to ensure that all promoter overlapping reads were included). Next, reads were subsampled with two parameters: the expected average coverage of the total mixture, and the expected prevalence of each underlying cell type. Given an expected average coverage, the expected total number of reads was calculated as (coverage * *domain size* / read length). Average read length was 100 bp, and domain size was estimated as the number of genes × 10 kb (the size of the TSS ± 5kb window). Next, the expected number of reads from each cell type was calculated as prevalence × total reads. Given the number of reads from each cell type, we calculated a sampling rate for each file, assuming single-end reads are present. This setup allows us to sample differences in coverage across each region and does not impose that more reads must come from the more prevalent cell type, which may not be true upon sequencing. Once a simulated mixture was generated, its processed methylation was obtained using the methRatio.py script provided with bsmap, and methylation data from both strands of DNA were consolidated for analysis. CpGs with fewer than 4 reads or more than 1000 reads were excluded from analysis. Similarly, read-level mixtures for enhancers were generated using the coordinates for the enhancer region instead of the window around the TSS.

### DMR calls

We utilized DSS (30) as an independent DMR-caller to help evaluate DXM results. DSS uses a Bayesian hierarchical model and was run using the following parameters: equal dispersion = True (for comparing single samples against each other), deltaVal = 0.3 (‘30% methylation difference’), and a pval threshold of 0.05 (a CpG is considered differentially methylated if this threshold is met).

### Identifying regions with distinct methylation signatures in mixtures of more than two cell types

Promoter regions (±5 kb around the TSS) were considered to have distinct methylation patterns if there was a DMR identified between each pair of cells considered. For example, for cell types A, B and C, a region is considered to have three distinct patterns if there is a DMR between A–B, B–C and A–C in the region.

### Region signatures for prevalence calling

Region signatures for prevalence calling were defined by first running DSS to identify DMRs between germinal center B cells (GCB, Blueprint: T14_11) and monocytes (Blueprint: S000RD13). Next, this list was further filtered for genes that showed differential expression (FPKM > 5 per sample, > two log-fold difference) between the two samples (Blueprint T14_11 and S000RD).

### MethylPurify and methylflow

MethylPurify (22) and methylFlow (36) were run with default settings except as noted below. For methylPurify, the most informative bins were identified from the ‘*.Informative_bins.bed.OneForCGI’ file, and subpopulation profiles were found in the MethylProfile.bed file. For the mixture of HMEC and HCC1954 cells methylPurify was run with default parameters. For GCB and monocyte mixtures, both CGI and ±5 kb around the TSS of all RefSeq genes were used as windows for the analysis, and the methylPurify code was altered to not filter reads ≥100 bp. For methylFlow, the methylation profiles of individual segments were obtained from the ‘patterns.tsv’ file, where pattern id (pid) refers to the number of profiles solved for a segment.

### Partial methylation in imprinted regions

All reads overlapping the region for at least 1bp were found using tabix (part of samtools). For reads overlapping the region boundary, only the part of the read that overlapped with the imprinted region were considered to determine its partial methylation status. If all CpGs covered by a read in the imprinted region were fully methylated or fully unmethylated, the read was considered to be non-partially methylated; otherwise it was labelled as partially methylated. Reads are equivalent to fragments since data are from single-end reads.

### Evaluation metrics

To evaluate whether DXM-solved methylation profiles resembled those of reference cell-types, we first computed the ‘closest possible’ reference methylation profile by rounding all fractional methylation values of the reference cell types to either 0 (fully unmethylated) or 1 (fully methylated). DXM-solved methylation profiles were then compared against each closest possible reference methylation profile and assigned to the reference that differed at the fewest number of CpGs (e.g. L1-norm difference). During assignment, DXM-solved subpopulations were not forced to be associated to different reference cell types, and assignment was allowed even in cases where there was significant discrepancy (e.g. >50% of CpGs).

Accuracy was reported as the percent of CpGs that were incorrect out of all CpGs for that gene promoter. After each profile was assigned to a reference cell type, to evaluate how accurate DXM was in identifying cell types at the correct relative prevalence, the major allelic subpopulation was defined from the expected prevalence of the underlying subpopulations in the mixture. The percent of major subpopulation profiles DXM identified that corresponded to the correct expected major cell type was then computed.

### Ontology analysis

Ontology analysis was conducted using the DAVID functional annotation tool with default parameters and background as *H. sapiens*.

### Primary samples

Primary DLBCL samples from lymph node biopsies were obtained from the WUSM Lymphoma Banking Program. Written informed consent was obtained from all patients as part of the WUSM Lymphoma Banking Program. This study was approved by the Washington University in St. Louis Institutional Review Board (#201710120). A full table of sample characteristics is provided in (Supplementary Table S1). Clinical flow cytometry was performed by the Barnes-Jewish Hospital clinical laboratory at the time of biopsy using standard clinical protocols for CD3+ T and CD19+ B cells. Sections of biopsy for tissue banking were processed to single cell suspensions and cryopreserved at >10 million cells/ml by the WUSM Tissue Procurement Facility. Samples were flash-thawed at 37°C and pelleted at 200 rpm, 5 min, 4°C. Samples were then resuspended in wash buffer (4% Fetal Calf Serum in dPBS). Half of each sample was isolated for Agilent-Methyl Seq, and the remaining half of each sample was sent for cell sorting.

### Cell sorting (FACS)

Each sample was first blocked with 100ul of 5% Human TruStain FcX (Biolegend) in wash buffer (4% FCS in dPBS) for 7min at room temperature. Samples were pelleted (200g, 5 min, 4°C) and resuspended in 100ul of the following binding buffer: 20ul (1 test volume) mouse anti-human CD19-PE (BD Biosciences), 20 ul (1 test volume) mouse anti-human CD4-FITC (BD Biosciences), 5ul (1 test volume) 7-AAD (BD Biosciences), and 45ul wash buffer. Samples were incubated in the dark at 4°C for 20 min. Following incubation, samples were washed twice with wash buffer before sorting with BD FACSAriaII at a standard flow rate for PE, FITC, and PerCP-Cy5.5-A channels. Standard FSC and SSC gates were used to identify lymphocytes. CD4+/CD19-/7-AAD- cells and CD19+/CD4-/7-AAD- cells were collected for subsequent analysis.

### Agilent methyl-Seq analysis

Genomic DNA was isolated from each sample using the Zymo Quick-DNA Miniprep kit. Agilent SureSelect Methyl-Seq was then conducted for each sample following manufacturer's recommendations for 1ug of input gDNA. Briefly, gDNA was sheared using a Covaris instrument at the recommended settings to an expected fragment size 150–200 bp, as confirmed by 2% agarose gel. Samples underwent end-repair, poly-A tailing, and ligation of methylated adapters to facilitate capture enrichment. DNA was resuspended in nuclease-free water as recommended and underwent vacuum-concentration to a volume of <5 ul. This methylated-adapter ligated DNA was then allowed to hybridize to the capture regions as per manufacturer recommendations. Next, bisulfite conversion was conducted on the input DNA at 64°C for 2.5 h using the EZ DNA Methylation-Gold kit (Zymo), After conversion, libraries were PCR amplified and then indexed using provided indices in the Agilent Methyl-Seq kit. Samples were sequenced with an Illumina Hi-Seq3000 (2 × 150 paired-end reads). Sequencing specifications are found in a table. On average, we obtained >50× coverage across ∼4.9 million CpGs for each patient, and we observed a minimum bisulfite conversion efficiency of >98.9% for all samples, as estimated by nonCpG conversion rate. All data were then aligned with biscuit v0.3.8 (https://huishenlab.github.io/biscuit/) and processed methylation data were obtained with the BISCUIT pipeline as recommended in the quick start page (pileup and vcf2bed commands). Data was collapsed per strand, and only CpG methylation data was considered.

### Targeted bisulfite sequencing

A table of all bisulfite sequencing primers and locations is provided in Supplementary Table S2. Bisulfite primers for loci of interest were designed using methPrimer2.0 (37) with default settings and BiSearch (38), requiring only one major genomic location for expected amplification. Primers were temperature-optimized for PCR amplification (QIAGEN Pyromark PCR kit) using bisulfite converted gDNA (as above) from GM12878 cells. gDNA was isolated and bisulfite converted for each sample as above. PCR-amplification was conducted for each region in each sample using 100pg of bisulfite-converted DNA as input. PCR products were barcoded and made into Illumina sequencing libraries as in (39). Libraries were sequenced on an Illumina Hiseq 4000. Sequences were trimmed for adaptors and poor-quality sequence using TrimGalore (https://github.com/FelixKrueger/TrimGalore) with default parameters. Sequences were then mapped to their target regions using Bismark (40) and default parameters and methylation levels (mCG/CG) extracted with bismark_methylation_extractor using the appropriate flag to collapse methylation across strands and otherwise default parameters.

### Enrichment analysis

To determine the enrichment of CpGs associated with i-DMRs in genomic elements (CpG island, shore, other elements), the observed distribution of CpGs in i-DMRs for these elements was compared to the expected distribution of CpGs in the TSS ±5 kb window that were detected by Agilent Methyl-Seq for these genomic elements. To determine the enrichment of iDMRs in the region around the TSS, we plot the log_2_-ratio of the observed normalized CpG counts in i-DMRs to the expected normalized CpG counts of CpGs for a given position relative to the TSS seen in either Agilent Methyl-Seq or WGBS.

### Statistics

Distributions were compared using Student's t-test for two groups or ANOVA with Tukey's posthoc *t*-test for multiple groups. Cohen's *d*-test was used to estimate effect size. Chi-square test for proportions was used to compare distributions of CpGs for enrichment analysis.

## RESULTS

### Features of methylation distributions in sorted cells

Before developing a deconvolution strategy, we sought to understand what constitutes distinct DNA methylation profiles in bisulfite-sequencing data both globally and in imprinted regions. We first examined the distribution of methylation levels (mCG/CG) within individual sorted cell types from the Blueprint Epigenome Project (BEP). One would expect that within a sorted cell type there would be relatively sharp peaks at 0%, 50% and 100% corresponding to unmethylated, imprinted or allele-specifically methylated, and methylated CpGs. However, from inspecting the distribution of methylation values, there are a variety of intermediate methylation values (i.e. not fully methylated or unmethylated) as well as substantial shoulders to the expected peaks around 0% and 100% methylation (Figure 1C). We next examined methylation in three types of blood leukocytes at imprinted loci. Imprinted loci are characterized by a DMR that is fully methylated in one inherited allele and fully unmethylated in the other (33,41). Despite this, we observe a substantial number of partially methylated reads as seen for the DMR near FAM50B in Figure 1D (other examples are in Supplementary Figure S2a). In fact, we find that on average 26.6% of bisulfite sequencing reads across DMRs in three hematological cells show partial methylation, or evidence for methylated and unmethylated CpGs on the same allele (Figure 1D, E, Supplementary Figure S2a). This results in a fractional methylation distribution at imprinted DMRs that is wider than what might be expected based on binomial sampling for a profile with 50% methylation (one methylated and one unmethylated allele, Supplementary Figure S2b).

These deviations from idealized distributions at imprinted domains, and fully methylated and unmethylated CpG sites likely do not arise from technical issues since both sequencing read quality (Supplementary Figure S2c) and bisulfite conversion efficiency are high (>99.7% overall reported for REP, (42), Supplementary Figure S2d). Moreover, local variation in bisulfite conversion efficiency in these regions does not drive the observed methylation variability seen in partially methylated reads (Supplementary Figure S2d, e). As such, these observations likely represent noise due to further biological heterogeneity (e.g. more than two underlying states) or biological noise such as age-associated drift (43) that is frequently smoothed to facilitate interpretation. In addition, the fractional methylation of a CpG across all reads appears to be insufficiently modeled by a binomial distribution defined by the number of reads and expected average methylation to model the counting errors due to sampling. Thus, as we developed our deconvolution strategy, we incorporated a beta-binomial distribution to model additional observed errors in the fractional methylation of each CpG across reads. The parameters of the beta-binomial are learned from real-sequencing data.

### Smoothing individual methylation profiles leads to a solution that is easier to interpret

We next applied methylFlow (MF) (36) to a simulated mixture of reads from whole-genome bisulfite sequencing of HMEC (Human Mammary Epithelial Cells) and HCC1954 (breast tumor) cells to understand whether interpreting every methylation change at each CpG as an individual pattern could lead to a useful result. methylFlow uses a network flow analysis to stitch together segments of methylation patterns and then combine these segments to identify underlying methylation profiles supported by bisulfite sequencing reads from an experiment. We focused on a wide window around the gene's transcription start site (TSS ± 5 kb) that is a conservative estimate for a region that likely includes the gene promoter and where methylation differences are most predictive of gene expression (44). From a 22× coverage simulated mixture of HMEC:HCC1954 (35:65) methylFlow identified 99.5% (17 367 of 17 450 ) gene promoters (TSS ± 5 kb) as having multiple methylation profiles (Figure 1F). For comparison, we only identified 6.9% promoters (1203 of 17 450) to have DMRs between the two cell-types. Within each promoter window, methylFlow found on average ∼55.7 profiles in the segment with the most potential profiles (Figure 1G). We also found that the log of the segment coverage is highly correlated with the log of the number of profiles in a segment (*r* = 0.75, Pearson) and the log of the segment length (*r* = 0.89, Pearson) (Supplementary Figure S3a, b). It is highly likely that this increase in patterns is driven by noise since the potential for individual read errors and biological noise increases with coverage as well.

Given that methylation across these regions is associated with expression (29) and that the functional significance of a methylation change at a single CpG site is usually unclear, it becomes difficult to interpret the number of potential patterns in each segment. Further, since the methylFlow segments are relatively small with a median length of 200–750 bp (36), enumerating the potential combinations of these profiles yields many more potential profiles at each promoter. As an example, there are upwards of 1.7 × 10^9^ possible promoter profiles from combining predicted segments for LARP4B across the 10 kb region centered at the TSS (Figure 1F). This corresponds to orders of magnitude more profiles than the number of cells used for methylation analysis. As such, though consideration of the spectrum of possible methylation profiles can be of interest, many researchers may want to interpret their data as an average methylation profile for a cell-type in a mixture. Based on the analysis of imprinted regions and this analysis, we thus sought to balance enumerating individual patterns with substantial smoothing to facilitate biological interpretation in our deconvolution strategy.

### Number of cell type-specific methylation patterns

An important consideration in any deconvolution strategy is how many different methylation patterns are expected in a complex mixture. To understand how many different methylation patterns between cell-types would be expected in mixtures of hematologic cell types, we identified all DMRs between nine different hematological cell types in large promoter windows (TSS ± 5 kb). Next, for a group of cell-types (e.g. GCB, CD4T, monocyte), we considered if the methylation profiles in a given promoter window could reliably distinguish among the cell types, defined as if there were at least one DMR between each pair of cell-types (see examples in Supplementary Figure S4) in the promoter window. To be as inclusive as possible, we did not merge any DMRs that overlapped multiple cell-types. Even using this set of criteria, which likely overestimates the number of genes with distinct DMRs between cell types, we found that in a given promoter window, there are rarely, if ever, four or more distinct DMRs between these cell types (Figure 1H).

### DXM design criteria

Based on these observations, we developed DXM to determine subpopulation methylation profiles spanning a user-defined set of genomic regions (e.g. a ±5 kb promoter window for expressed genes, CGIs, imprinted DMRs, etc.) and a list of intrasample differentially methylated regions (i-DMRs), or regions where allelic subpopulations have differential methylation. Each region is solved separately, reflecting how the number of distinct allelic subpopulation methylation profiles will vary across different regions (e.g. in a mixture of three cell-types, each promoter could have 1–3 different methylation profiles). DXM considers every CpG in a region for calculation but offers an effective method of smoothing out smaller individual changes without using binning. The resulting methylation profiles solved by DXM are binarized, reflecting how a given allele is either methylated or unmethylated.

### DXM performance on simulated idealized mixtures

We first demonstrated DXM performance in simulated idealized cases of HMEC-CD4+T and HMEC-HCC1954 cell mixtures. To remove potential noise, methylation levels (mCG/CG) were binarized to 0 and 1 for each cell type. For a fixed coverage, unmethylated and methylated counts for each CpG site were modeled using a binomial distribution. This effectively assumes that errors from inadequate bisulfite conversion and sequencing are negligible relative to the binomial sampling error (see methods for details). As seen in these simulations, DXM performs very well. The number of genes detected to have multiple profiles (i.e. sensitivity) increases with coverage until ∼30–90× depending on the prevalences of the underlying cell-types (Supplementary Figures S5a and S6a). The accuracy of each profile is very high across most coverages (see Evaluation Metrics in methods for details, Supplementary Figures S5b–c and S6b–c), and on average 96.2% for HMEC-CD4+T cell mixtures and 95.1% for HMEC-HCC1954 cell mixtures when the coverage is greater than 30×. Together this demonstrates that DXM is generally optimized to only call profiles when it can do so accurately, otherwise it refrains.

### DXM identifies allele-specific methylation profiles in X-linked CGIs and imprinted regions

**Figure 2.**
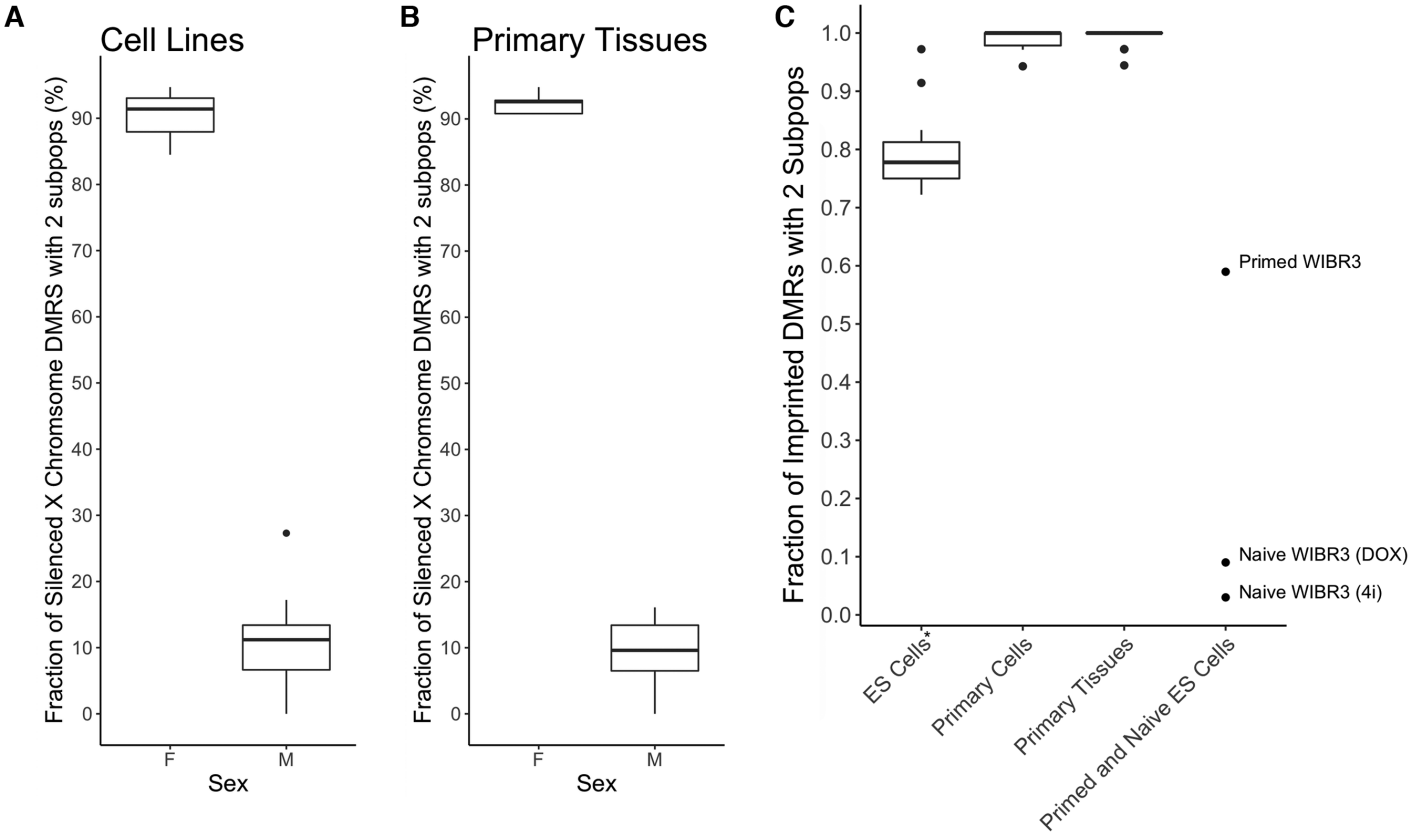
DXM accurately deconvolves methylation profiles at X-linked CGIs and imprinted regions. (A, B) DXM was used to deconvolve CGIs identified on the X chromosome in (**A**) cell lines and (**B**) primary tissues. DXM finds multiple methylation patterns in a median of 91.4% of female cell lines and 92.6% of female primary tissues but only in 11.2% male cell lines and 9.6% in male primary tissues. (**C**) DXM accurately deconvolves methylation profiles in imprinted regions. DXM was used to deconvolve CGIs identified on the X chromosome in cell lines and primary tissues from the Roadmap Epigenomics and Blueprint projects. ES Cells* include ES cells, ES derived cells, and iPSCs, which are known to lose imprints. DXM accurately predicts two subpopulations in imprinted regions for 99.0% (485 out of 490) of imprinted DMRs with average coverage ≥10 in primary cells and at 99.3% (751 out of 756) in primary tissues. DXM finds fewer patterns of imprinting (∼76%) in ES cells as expected. Full lists of the samples used are in Supplementary Tables S3 and S4. Data from primed WIBR3 ES cells as well as naïve WIBR3 cells converted using 4i/L/A or DOX (doxycycline-inducible *KLF2* and *NANOG* transgenes) is from (28).

To determine whether DXM could identify allele specific methylation profiles in somatic cells, we next considered X-linked CGIs and imprinted regions. We first applied DXM to deconvolve methylation data from CGIs associated with X-inactivated genes in 14 cell lines and 21 somatic tissues (Figure 2A, B, Supplementary Table S3). On average, we identified differential methylation patterns across ∼92% of the CGIs in females but only ∼14% of the CGIs in males. The variance from the hypothetical result of 0 and 100 is within what is expected due to a normal variation (e.g. age-associated drift) or sex-specific methylation patterns in males (45). For imprinted regions, we first analyzed methylation profiles of imprinted regions at various somatic tissues and cell types (available at REP and BEP, Supplementary Table S4). DXM accurately predicts 2 subpopulations in imprinted regions for 99.0% (485 out of 490) of imprinted DMRs with average coverage ≥10 in primary cells and at 99.3% (751 out of 756) in primary tissues. We then used DXM to analyze imprinted regions in the human embryonic stem (ES) cell line WIBR3 before and after converting it to a naïve state using 4i/L/A media or doxycycline (DOX)-inducible KLF2 and NANOG transgenes (28). DXM identified multiple methylation patterns at 59% of imprinted loci for primed WIBR3 cells, and at less than 10% of imprinted loci for naïve WIBR3 cells (Figure 2C, Supplementary Table 4). This is in concordance with the expectation that ES cells lose imprints when converted from primed to naïve states (28). Taken together, these results demonstrate the utility of DXM analysis to examine allelic methylation patterns in the context of X-inactivation and genomic imprinting.

### DXM accurately solves subpopulation methylation profiles in heterogeneous mixtures

**Figure 3.**
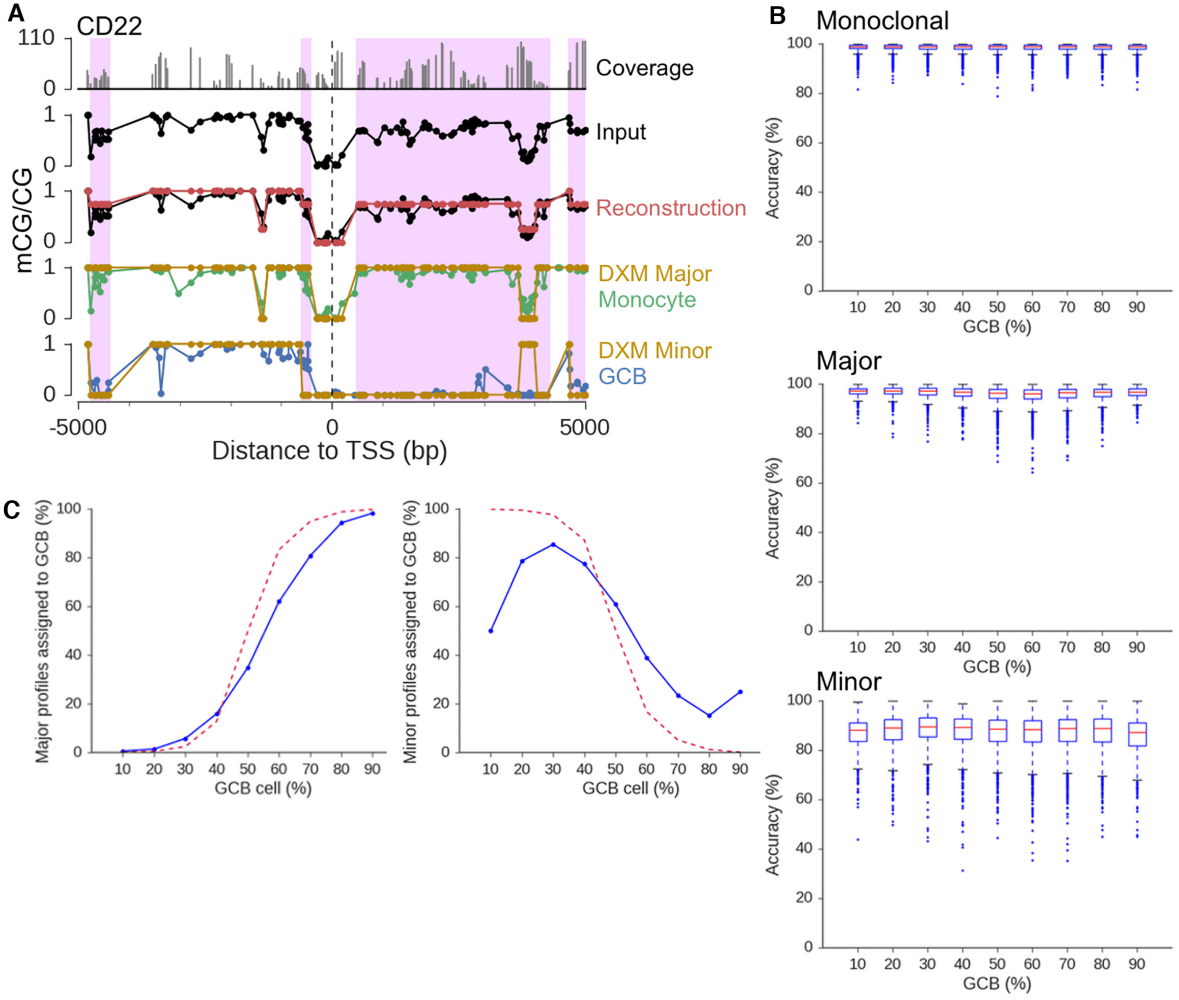
DXM accurately solves for subpopulation methylation profiles in heterogeneous mixtures. (**A**) DXM solution for CD22 for a 55× coverage simulated mixture of monocytes and GCB (35:65). (**B**) Accuracy of DXM methylation profiles with respect to reference GCB or monocyte profiles for promoters where DXM identified the same number of methylation profiles as expected by DSS. (**C**) Number of gene promoter profiles assigned to GCB cells (blue) across 55x coverage mixture simulations with different GCB prevalences. The dotted red line indicates the number of CpGs with more subsampled reads from GCB cells than monocytes.

To determine the effectiveness of DXM in solving deconvolved methylation profiles in cellular mixtures, we generated a series of simulated mixtures by subsampling bisulfite sequencing reads from sorted germinal center B-cells (GCB, from tonsil (24)) and monocytes (from cord blood (24)) at a fixed expected average coverage of 55×. Our simulations included cases where there were more GCBs as well as more monocytes. We initially considered methylation profiles in a ±5 kb window around the TSS since this includes the regions where differential methylation most likely impacts gene expression (44). We then applied DXM to deconvolve these mixtures (example output in Figure 3A, Supplementary Figure S7) to assess performance.

We first evaluated the accuracy of DXM-deconvolved subprofiles. Methylation profiles output by DXM were highly accurate for both major and minor subpopulations. DXM correctly identified methylation across 98.5% of CpGs for promoters with one methylation pattern, 96.2% for major methylation profiles, and 87.1% for minor methylation profiles (average performance across all prevalence mixtures for GCB-monocytes, Figure 3B). Similar results were obtained for mixtures generated from sorted CD4+T-CD8+T cells from BEP (Supplementary Figure S8) as well as from a mixture of HMEC and HCC1954 cells (Supplementary Figure S9). Increasing sequencing coverage up to 88x did not substantially impact reconstruction accuracy (Supplementary Figure S10a) or profile assignment (Supplementary Figure S10b), consistent with earlier simulations (Supplementary Figures S5 and S6).

We next examined whether DXM deconvolved profiles could be assigned to the appropriate cell type at promoter regions with distinct cell-type specific methylation profiles (i.e. promoter regions that contain a DMR identified between the two cell types). For each promoter, DXM-solved subpopulation methylation profiles were assigned to the closest reference cell type. As expected, the percent of profiles assigned to the GCB cell type in the major subpopulation increases as the ratio of GCB reads increases (Figure 3C). The inverse relationship can be seen for the minor subpopulation. DXM performs best when the minor subpopulation comprises 20% or more of the mixture. This might be expected for our simulations, since in a 55× coverage mixture, a 10% subpopulation corresponds on average to only 5.5 reads. For some promoters, especially when the prevalence of the minor subpopulation is less than 20%, the minor methylation profiles output by DXM are closer to the true major subpopulation reference profile, rather than the minor population reference (Supplementary Figure S11a, b). This observation likely reflects how intrinsic variation in typical methylation data from single sorted cell types at fully methylated and unmethylated CpG sites, the purity of the sorted cell populations, and the number of CpGs with differential methylation between cell subpopulations masks the ability of deconvolution approaches to detect low prevalence subpopulations (Figure 1C-E). Even with the beta-binomial error models used by DXM, noise in individual datasets can substantially affect the smallest allele fraction that can be detected.

### DXM predictions for more than two subpopulations

We further sought to evaluate DXM performance for mixtures with more than two subpopulations. We applied DXM to a 55× simulated mixture of CD4^+^T:GCB:monocytes (10:25:65). We focused on only 3 subpopulations since we found very few gene promoters with more than that many distinct profiles (Figure 1H, Supplementary Figure S12a). The 496 genes that have a DMR between each pair of cell types in this mixture showed enrichment in several immune pathways, which is expected from ontology analysis. DXM identified multiple methylation profiles at 434 of the 490 (88.5%) promoters expected to have three methylation profiles, but 342 (79%) of these promoters were solved with only two profiles instead of three (Supplementary Figure S12b). This suggests that in those 342 promoters, DXM identified subpopulation methylation differences but could not resolve them into three distinct profiles. For the deconvolved promoters, DXM had high accuracy for both the major (95.9%) and minor (87.4%) profiles (Supplementary Figure S12c). This accuracy resembles what was seen for *k* = 2 simulations (Figure 3B). For the 57 promoters solved with three methylation profiles, DXM had high accuracy for the first (96.0%), second (87.0%) and third (77.6%) profile (Supplementary Figure S12d). Given that on average only 5.5 reads are supporting the lowest subpopulation, it is thus not surprising that DXM frequently does not detect this subpopulation, and when it does it has lower accuracy. Taken together, DXM can accurately deconvolves subpopulation methylation profiles in mixtures with three subpopulations.

### DXM accurately deconvolves subpopulation methylation profiles at enhancers

We applied DXM to a list of enhancer regions defined based on H3K27Ac and H3K4me1 ChIP-Seq data that are expected to be active in either GCB cells or monocytes (46,47). We performed similar read-level simulation mixtures to those above and found that DXM performed well with median reconstruction accuracies across all prevalences of >99% if the same methylation pattern is present, 96.1% for the major subpopulation, and 80.0% for the minor subpopulation (Supplementary Figure S13). This performance is maintained across all mixture conditions. This suggests that the mathematical framework of DXM is sound, and that it can be applied to other genomic regions such as enhancers.

### DXM outperforms existing bisulfite sequencing deconvolution algorithms

**Figure 4.**
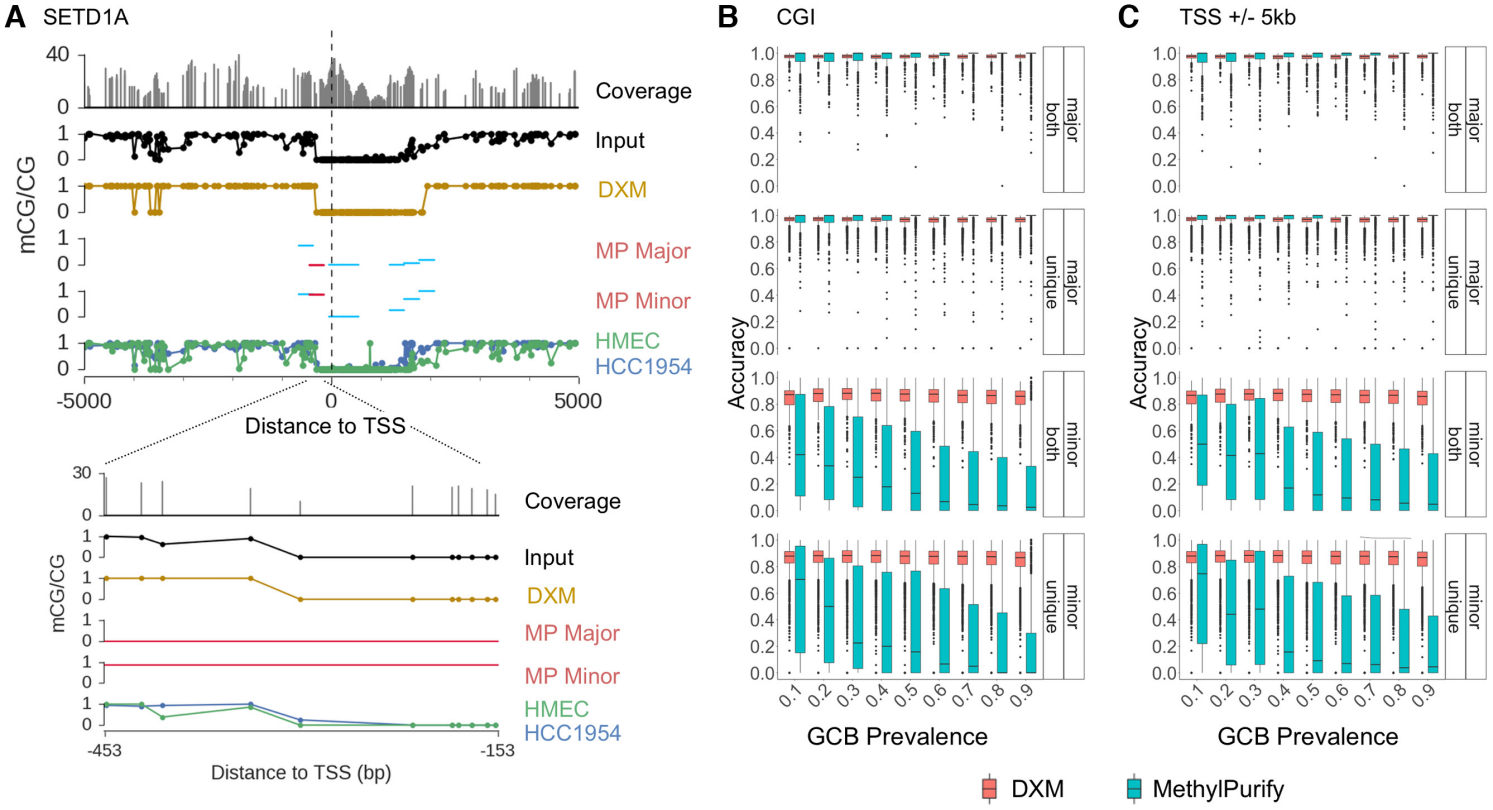
DXM outperforms existing methods. (**A**) methylPurify (MP) and DXM outputs for SETD1A in a 20× coverage HMEC-HCC1954 mixture (35:65). Both all (sky blue) and most informative (crimson) bins as determined by MP are depicted. Despite agreement between HMEC and HCC1954 profiles, MP separates this region into two profiles (likely due to the fixed bin size). DXM, however, predicts only one profile. (B, C) Reconstruction accuracy is plotted for (**B**) CGIs and (**C**) promoter windows (TSS ± 5 kb). Regions where both methods identified multiple methylation profiles are labeled ‘both,’ and regions where only one method identified multiple methylation patterns (DXM i-DMR, methylPurify informative bin) are plotted as ‘unique.’ Whether or not a region was identified by ‘both’ methods or by one method alone (‘unique’) did not affect accuracy. While DXM accurately deconvolves methylation profiles across CGI and promoter windows for both major and minor subpopulations, methylPurify only accurately deconvolves profiles for the major profile.

We compared DXM with methylPurify (22), an expectation maximization-based approach developed to separate normal and tumor cell profiles from a heterogenous mixture. MethylPurify first identifies a series of most informative 300 bp bins that show intermediate methylation. Using those bins, it then predicts the prevalence of the two underlying cell types and then estimates the average methylation in each cell type across that bin. Data from adjacent bins can then be stitched together to make a profile. MethylPurify has several limitations relative to DXM: it is only compatible with human genome versions hg18 and hg19, it appears to have been only benchmarked at CGIs, it assumes there are only two underlying cell types, and does not natively work with reads that are 100 bp or longer. MethylPurify also cannot distinguish methylation changes at individual CpG sites since it uses a binning scheme (default of 300 bp) to average all methylation changes within a bin (example in Figure 4A). We first generated a 20× coverage simulated mixture of HMEC:HCC1954 cells (35:65) and applied DXM and methylPurify to deconvolve the sample. We chose this data since it was used to originally benchmark methylPurify, and because the GCB:monocyte mixtures used above had longer read lengths and would not natively run with methylPurify. For this mixture, both methylPurify and DXM accurately solved methylation profiles of the underlying cell types (methylPurify 98.9%, DXM 97.8%). DXM identified i-DMRs at 58% (15 009/26 078) of the most informative bins (mib) predicted by methylPurify, while regions specific to either method tended to cover fewer CpGs (*P* < 0.001, Cohen's *d* > 0.8, Supplementary Figure S14a, b).

Next, we edited methylPurify's code to allow reads longer than 100 bp and compared DXM with methylPurify across mixtures of GCBs and monocytes where coverage was fixed at 40x (Figure 4B, C). While the accuracy of DXM and methylPurify is similar for the major subpopulation, DXM’s accuracy for the minor subpopulation is much higher. We found that DXM performs well across CGI and promoter windows, with average accuracy of ∼98% in major subpopulations and ∼84% across minor subpopulations. methylPurify does well with major subpopulations (average of 99% accuracy), but performance across minor subpopulations falls below 50% in most mixtures. MethylPurify had very low accuracy in regions identified by both it and DXM, suggesting the difference is not strictly due to methylPurify trying to deconvolve a more difficult set of regions. We suspect methylPurify may underperform because it misestimates the fractions of the cell populations for these mixtures; it estimated that the prevalence was 0.055 for the minor cell type for most mixtures (Supplementary Figure S14c). Taken together, we conclude that while DXM performs similarly to methylPurify at accurately deconvolving DNA methylation profiles for major subpopulations, DXM is more robust at accurately deconvolving the minor profile across a variety of types of cell mixtures and regions.

### Given an appropriate region signature, DXM accurately solves prevalence of underlying cell-types in heterogeneous mixtures

While the primary goal of DXM was to deconvolve subpopulation level methylation changes, we next sought to determine whether DXM could also accurately estimate the prevalence of allelic subpopulations. The analogous problem for calling genetic subclones is based on the general assumption that genomic sequencing analysis of a sample containing a subpopulation with 20% prevalence should yield a peak in the distribution of variant allele frequencies (VAFs) around 20%, which reflects the mutations associated with the subclone. Similarly, if there are CpGs that only are methylated in a certain subclone that comprises 20% of the sample, the expected fractional methylation of these CpGs in the sample would be 20%. However, in a 20:80 mixture of GCBs and monocytes, we found that the methylation distribution of all CpGs does not even have a minor peak near 20% (Supplementary Figure S15a). Thus it would be difficult in practice to consider all CpGs to determine absolute prevalence. Instead the better approach is to select a subset of CpGs that differ between the clones and choose those. We first considered only CpGs that were found in i-DMRs (Supplementary Figure S15b), and found that the distributions still did not show clearly defined peaks around 20% and 80%, and in practice prevalence estimates were poor (data not shown). We next defined a signature based on gene promoters containing DMRs that are differentially expressed between GCB cells and monocytes in a different set of samples (see methods). Using this region signature, DXM predictions have very high agreement with expected prevalence when the expected prevalence of the minor subpopulation is between 15–40% (Supplementary Figure S15c, d). This suggests that given an outside signature of regions that show differential methylation between samples, DXM can accurately identify subpopulation prevalences.

### Comparison of i-DMRs from simulated mixtures and DMRs between cell types

We next examined the degree of concordance between DXM i-DMRs predicted from simulated cell mixtures and DMRs identified from the underlying cell types using the same GCB-monocyte mixtures as above. Our goal was to determine if DXM was reasonably calling i-DMRs. DXM on average found an i-DMR between underlying subpopulations in about 64% of all DMRs (Supplementary Figure S16). This is consistent with what would be expected given the wide-variability seen in different DMR callers (48–50). Across all simulated mixture ratios, we found that DXM predicted a much larger number of i-DMRs for GCB-monocyte mixtures relative to the number of DMRs DSS identified between each underlying cell type (e.g. for a 30:70 mixture, 19 755 i-DMRs and 3012 DMRs, or 5722 versus 2187 gene promoters).

The DMRs that were not identified by DXM tended to be shorter and have fewer CpGs (Supplementary Figure S17). Shorter DMRs with fewer CpGs have less empirical support and whether they are called DMRs tends to be more variable across DMR callers. Additionally, we found that increasing sequencing coverage did not improve this overlap, though it increased the total number of i-DMRs and DMRs found (Supplementary Figure S18a). However, given the coordinates of where to expect these DMRs, we next isolated each ‘missed’ DMR and reran DXM for just the DMR instead of across the full ±5 kb promoter window. With this updated region definition, we found that on average, DXM identified as i-DMRs ∼86% of these previously ‘missed DMRs’ (Supplementary Figure S18b). Taken together, this highlights how DXM and DSS use different approaches to smoothing the data and locating DMRs but can recapitulate similar results.

### DXM identifies substantial subpopulation methylation profiles in sorted cell types

**Figure 5.**
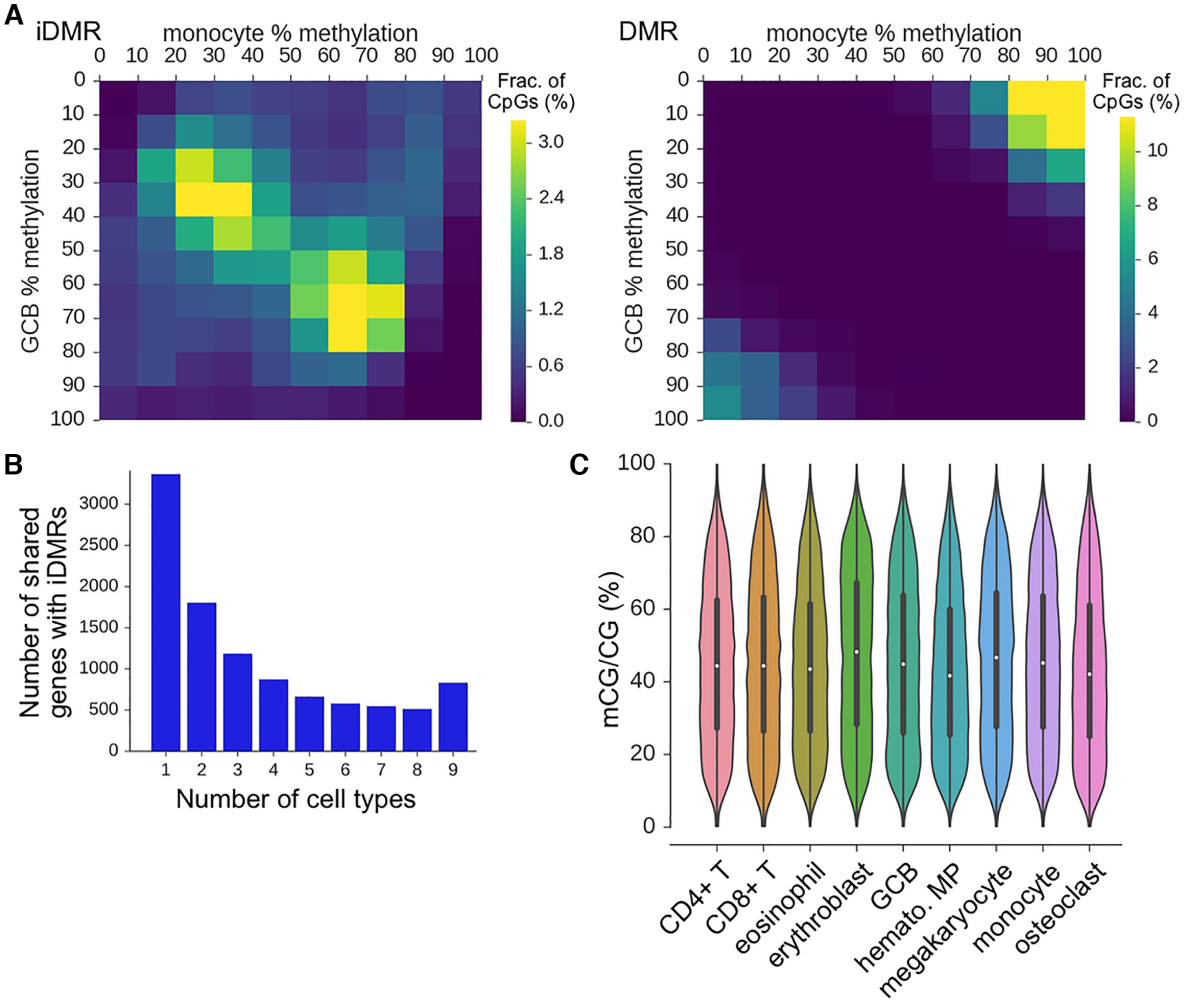
DXM identifies many i-DMRs within sorted cell types. (**A**) i-DMRs and DMRs identified in a 55× mixture of GCB:monocytes (30:70) have different methylation profiles of the underlying reference GCB cells and monocytes. For instance, many i-DMRs are found with 30–40% methylation in both GCB and monocytes. (**B**) The number of gene promoters with i-DMRs that are shared between different numbers of sorted cell types is shown. There are substantial numbers of i-DMRs that are both unique to individual cell-types, as well as shared across many cell types. The cell types used are listed in (C). (**C**) Fractional methylation distribution of CpGs in i-DMRs for sorted cell types.

We next sought to understand why DXM predicted many i-DMRs in regions that did not have a DMR between the cell-types. Plotting the distribution of methylation levels (mCG/CG) for the reference GCB and monocytes shows that these levels are often similar between cell-types but in an intermediate methylation state (e.g. 30% methylation in GCB and 30% in monocytes) (Figure 5A). One interpretation is that for these loci, there are distinct DNA methylation patterns in subpopulations, but these subpopulations differ from the expected reference cell types as might be defined from sorting. Supporting this is the observation that when we applied DXM to only GCB cells or only monocytes alone, we found that 70% of the DXM-specific i-DMRs predicted in the GCB:monocyte mixture were also detected as i-DMRs within only GCB cells or only monocytes. However, it is unlikely that the multiple patterns found within these cell types represent what is traditionally referred to as biological heterogeneity (e.g. multiple cell subtypes within a sorted cell type such as light and dark zone GCBs or classical/non-classical monocytes), since the sub-profiles are mostly shared by two distinct sorted cell types: GCBs and monocytes. For instance, when we look at i-DMRs that lie within nine total sorted cell types (CD4+ T, CD8+ T, eosinophil, erythroblast, GCB, hematopoietic multipotent multiprogenitor, megakaryocyte, monocyte, osteoclasts), we detected 840 promoters with an i-DMR within each of the nine cell-types and ∼500 genes where five cell-types exhibited the i-DMR (Figure 5B). The distribution of methylation levels of CpGs in these common i-DMRs is not centered around 50%, suggesting that they are likely not due to imprinting or allele specific methylation (Figure 5C).

We next sought to verify that the extensive heterogeneity we observed was likely due to multiple cells in these sorted populations with distinct biological states. We thus ran DXM on individual sorted cell types amongst nine hematologic cell-types (CD4T, CD8T, eosinophil, erythroblast, GCB, hematopoietic multiprogenitor cells, megakaryocytes, monocytes, osteoclasts). Ontology analysis indicates that i-DMRs that are shared across all nine cell-types are enriched for genes associated with cadherin-domain proteins (Supplementary Table S5). Cadherins are critical to cell-adhesion (51), which regulates many aspects of leukocyte function, including extravasation and vascular permeability for circulating leukocytes (52). This suggests there is a methylation signature across these genes that could be associated with reduced cadherin expression, which could reflect decreased tendency of a particular cell to adhere or a change in active state for the cell (e.g. circulating vs extravasating). Further, because cell-adhesion is a pathway common to all cells, this is consistent with the finding that there are multiple methylation profiles within sorted cell types that may not correspond to traditional cell subtypes but certainly could represent distinct biological states. Thus, DXM-specific i-DMRs likely represent a separate class of relevant subpopulation methylation events.

### DXM predicts subpopulation differences in methylation in primary DLBCL samples

**Figure 6.**
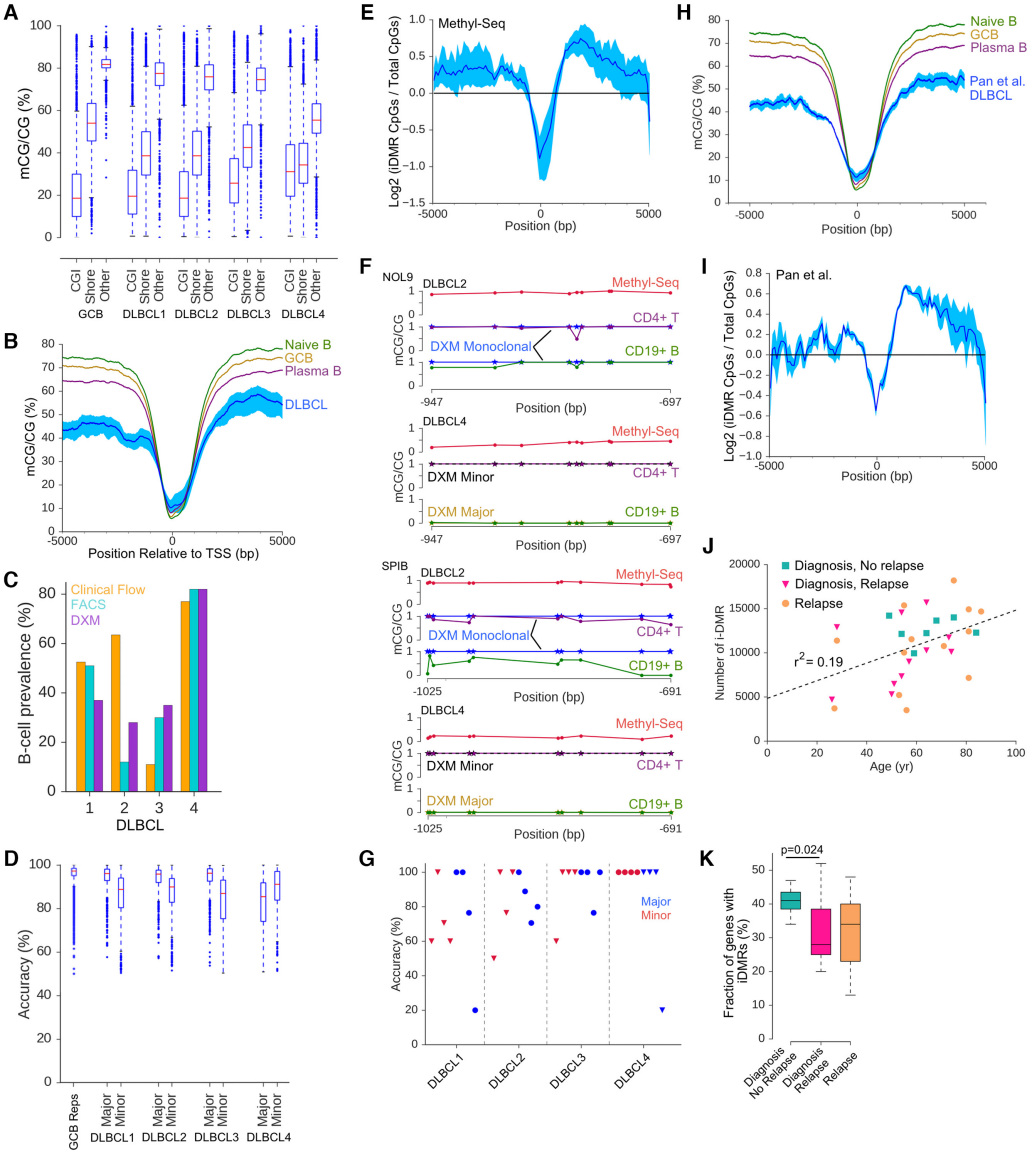
Experimental validation of DXM predictions in DLBCLs. (**A**) Distribution of methylation for CpGs in CGIs, CGI shores or other regions for four DLBCL lymph node biopsies profiled by Agilent Methyl-Seq. (**B**) Meta-gene analysis (with respect to the TSS) of the four DLBCL samples and reference cell types show hypermethylation at and hypomethylation up- and down-stream of the TSS. Dark blue denotes the average sample profile, with light blue indicating the maximum and minimum range. (**C**) Percentage of B cells as measured by clinical flow (orange), FACS (light blue), and DXM (purple). (**D**) Accuracy of DXM predictions for each sample at 2454 gene promoters with DMRs identified between normal CD4^+^ T-cells and GCB cells. (**E**) i-DMRs are enriched 500 bp–3 kb downstream of the TSS. Shading as in B. (**F**) Targeted bisulfite sequencing results for *NOL9* and *SPIB* for two DLBCL samples. Results for additional genes and samples are in Supplementary Figure S19. Methyl-Seq input data (red), DXM predictions (monoclonal blue, major profile gold, minor profile black dash), sorted CD4+ T-cells (purple), sorted CD19+ B-cells (green). (**G**) Accuracy of DXM predicted profiles in a targeted bisulfite sequencing experiment for four DLBCL samples across four loci (*NOL9*, *SPIB*, *CD22*, *BCL2L1*) for sorted CD19+ B-cells and CD4+ T-cells. Blue = major subpopulation, Red = minor subpopulation, triangle = B-cell, circle = T-cell. (**H**) Meta-gene analysis (with respect to the TSS) of the 31 DLBCLs from Pan *et al.* (5) and reference cell types show hypermethylation at and hypomethylation away from the TSS. Shading as in B. **(I)** i-DMRs are enriched 500 bp–3 kb downstream of the TSS in 31 samples from Pan *et al.* Shading as in B. (**J**) Number of i-DMR detected in the 31 DLBCL samples with respect to patient age (*r*^2^ = 0.19). (**K**) Fraction of gene promoters with an i-DMR for DLBCL samples at diagnosis (blue-green, *n* = 11), at diagnosis that did not have future relapse (pink, *n* = 7), and at relapse (orange, *n* = 13).

We next experimentally validated DXM predictions from Agilent Methyl-Seq analysis of lymph node biopsies from four DLBCL patients (sample characteristics are in Supplementary Table S1). Single cell suspensions from biopsies were split in half. Half of the cells were sorted by fluorescence activated cell sorting (FACS) into CD4+ T and CD19+ B cell populations, the expected major cell types present in DLBCL lymph node biopsies. The other half was subjected to Agilent Methyl-Seq, which is a bisulfite sequencing approach that enriches for gene promoter regions using capture probes similar to exome capture techniques (53). We obtained an average of >54x coverage across ∼4.8 million CpGs for all samples, including >58× coverage for >2 million out of ∼3.5 million CpGs in the 10kb region around the TSS (Supplementary Table S6). CGIs and the ±5 kb region at the TSS were hypermethylated in DLBCLs relative to normal cell types, while proximal regions were hypomethylated as has been observed in prior DLBCL studies (5) and are typical in most cancers (54) (Figure 6A, B).

After running DXM on each DLBCL sample, we first compared DXM-solved estimates (using a gene signature from normal CD4^+^T and GCB cells as above) of the relative proportion of T and B cells, to T and B cell proportions determined by FACS and to clinical flow cytometry. Of note, clinical flow cytometry for T and B cells was performed on an adjacent section of the same lymph node at the time of biopsy, while FACS was performed from the same single cell suspension used for Agilent Methyl-Seq. We found good agreement between DXM-estimated prevalence and FACS results (on average, fractional prevalence estimates were within 0.0875 of each other, B-cells shown in Figure 6C). Clinical flow data was consistent with FACS and DXM for all but one DLBCL sample (DLBCL 2). DXM results likely are more consistent with FACS sorting because clinical was obtained from a different section of the biopsy, and DLBCL lymph nodes are known to be heterogeneous.

Next, we estimated the accuracy of DXM subpopulation methylation profiles for the gene signature between normal CD4^+^ T-cells and normal GCB-cells. For these 2,454 genes, DXM identified differential methylation in 901–1141 promoters with an average accuracy of 91% for major subpopulations and 86% for minor subpopulations, which approaches the levels observed during benchmarking (Figures 3B and 6D). The clear outlier is DLBCL 4, which has substantially more B cells than T cells (Figure 6C) and shows greater CGI hypermethylation. Thus, the major population of DLBCL 4 is likely comprised of malignant B-cells, whose methylation profiles differ substantially from the normal GCBs used as a reference to estimate the accuracy, causing an artificially low estimated accuracy. For each sample, DXM found 3785–6790 gene promoters with at least one i-DMR (Supplementary Table S6). While i-DMRs were not enriched in CGIs or CGI shores (mean O/E = 1.01 for CGIs and 0.94 for CGI shores, *P* = 0.976), we did observe more i-DMRs in the region located 500–3000 bp downstream of the TSS (Figure 6E), which has been found to strongly associate with expression changes (29).

To experimentally validate DXM profile predictions, we conducted targeted bisulfite sequencing in sorted CD4^+^ T-cells and CD19^+^ B-cells from each sample. We selected four loci that regulate B-cell specific function (SPIB (55), CD22 (56)) or are commonly mutated in DLBCL (BCL2L1 (57), NOL9 (58)) and for which DXM predicted an i-DMR in at least one but not all samples. DXM predicted the correct number of underlying methylation profiles (one or two) across each locus and each patient (example in Figure 6F, data for all 16 cases found in Supplementary Figure S19). Additionally, DXM predictions for subpopulation methylation profiles are recapitulated in sorted cell types with an average accuracy of 84.7% (Figure 6G).

As proof-of-concept, we applied DXM to a publicly available cohort (Cohort 1 in (5)) of 31 DLBCLs (11 paired diagnosis-relapse, 1 of which had 3 relapses, 7 with no relapse) profiled by enhanced reduced representation bisulfite sequencing (eRRBS). These samples exhibit a similar gain of methylation at the TSS and loss of methylation outside the 2 kb surrounding the TSS (Figure 6H). DXM identified 3708–18 200 i-DMRs (average of 10 826) for these samples (Supplementary Table S7). i-DMRs were frequently found 500–3000 bp downstream of the TSS (Figure 6I) as seen with our DLBCLs (Figure 6E). We observed a weak correlation between age and the number of i-DMRs detected for each sample (Figure 6J, *r*^2^ = 0.19). This is suggestive that some i-DMRs may be caused by age-associated drift in DNA methylation patterns (43). Additionally, we found that patients presenting with fewer gene promoters with i-DMRs at diagnosis had higher rates of relapse (Figure 6K).

## DISCUSSION

We present DXM, a computational method to deconvolve methylation sequencing data from a heterogeneous sample into its major allelic subpopulations and their associated methylation profiles. Importantly, DXM does not require explicit prior knowledge of the expected cell types or number of subpopulations to consider. When using DXM, one consideration is that DXM solves for allelic profiles, which we then interpret as corresponding to unique subpopulations. As such, care must be taken in interpreting DXM results in regions with allele-specific methylation or copy number variants. The same is true for any deconvolution strategy. An additional consideration is that DXM does not consider 5-hydroxymethylation (5hmC), which may impact interpreting results in samples with high levels of 5hmC, such as neuronal cell types (59).

If given an appropriate set of regions that are known to show variation in the cell types, DXM can accurately measure the allelic fraction of each cell-type. One potential advantage of using DXM to detect cell prevalences is that DNA methylation is highly stable even in cryopreserved samples, especially relative to RNA and protein. Further, malignant cells are often more susceptible to cell death caused by delays related to specimen transport and cryopreservation (Payton, unpublished observations), which could potentially affect clinical flow and FACS. However, additional future testing is required to determine whether a methylation-based strategy using DXM detects cell prevalences more accurately than these standard approaches. As a pseudo-reference-free method, DXM does not require reference profiles for each cell-type. Instead, DXM uses generalized model parameters to identify subpopulation level methylation profiles. However, if reference profiles are available, they can still be useful to interpret DXM results.

To ensure we did not overfit DXM model parameters, we used independent datasets for all training and evaluation steps. While we have demonstrated that DXM can accurately deconvolve DNA methylation profiles for a variety of genomic features in the case studies above, it is possible that retuning DXM parameters (e.g. transition probabilities) for genomic compartments or for individual datasets could improve accuracy. This is likely true for samples with extreme methylation changes such as caused by mutation in a DNA methyltransferase (e.g. DNMT3A in an AML patient (60) or treatment with demethylating agents (61)).

A major challenge of genomic methylation data analysis is determining what constitutes a biologically meaningful methylation change, or a distinct methylation profile. DXM takes an approach to regularize small differences in methylation between two profiles to report a small number of smoothed profiles. This interpretation is used frequently for imprinting control regions, where partially methylated alleles are considered as biological noise, or drift from a true pattern rather than a functionally different state. A similar assumption underlies DMR-callers. The differences between DSS-called DMRs and i-DMRs in artificial mixtures are similar to those observed between different DMR-callers (62). DMR-callers rely on several important user-defined parameters such as the minimum length, minimum number of CpGs, or minimum difference in methylation between samples, that can severely affect the number and type of DMRs in an analysis (30). Despite this, DMR callers are incredibly useful and are part of every common workflow for DNA methylation analysis. The same should be true for DXM i-DMRs.

DXM deconvolves methylation profiles over user-defined regions. Here we have demonstrated how these regions could be CGIs, promoters, enhancers, or imprinted DMRs, but in theory DXM could be applied to any set of genomic regions. When defining regions however it is important to consider how region size will affect the results. As regions get larger and include more CpGs, the number of CpGs that must have a change in methylation in order to be solved as a distinct methylation profile by DXM will also increase. This can be seen by comparisons between DXM and DSS, where many of the DMRs identified by DSS were recapitulated by DXM when given a more specific domain to consider. This can be offset by increased sequencing depth, as the ‘resolution’ (number of methylation profiles solvable by DXM) of that region increases with increased coverage. While it is possible that with sufficient depth one could try to solve a global methylation profile across an entire chromosome, this would be difficult in practice given gaps in most genome assemblies and frequent large repetitive DNA sequences (e.g. simple repeats or transposons) that complicate obtaining high quality methylation data.

One surprising result from this analysis is that even sorted cell types have a substantial number of intrinsically intermediate methylated regions, or genomic intervals that are not fully methylated, unmethylated or imprinted. Intermediate methylation states can be both cell-type specific and conserved (63). Our results show that even in sorted cell types there is extensive intermediate methylation. One contributing factor is that cells are sorted on a small number of markers and likely contain multiple subsets within them. However, the substantial number of partially methylated reads in imprinted DMRs suggest that this is not the only contributing factor. This extensive intermediate methylation complicates deconvolution efforts, particularly reference-free cell prevalence determination. This suggests that there are fewer CpGs whose methylation is ‘cell-type specific’ as compared to CpGs with intermediate methylation levels in an individual cell type.

The origin of this intermediate methylation is not completely understood but likely includes several previously described phenomena including lowly methylated regions (LMRs) and partially methylated domains (PMDs). Initial reports have demonstrated that LMRs, relatively short non-CpG island DNA segments with low levels of methylation, frequently arise from the binding of transcription factors (64,65). However, we find near equal levels of highly and lowly methylated DNA methylation in i-DMRs indicating that while LMRs could contribute to the i-DMRS we observe in individual cell types, they cannot fully explain them. PMDs are broad regions of lower methylation, are commonly found in cancers and to a limited degree in somatic cell types, and are associated with hypervariability (66). Cell-type specific PMDs are predicted by CpG density in addition to late replicating/lamina associated domains (67). Given the lack of association of i-DMRs with CpG-density, PMDs could contribute to the i-DMRs but are also unlikely to explain them all.

Lastly, we have validated that subpopulation methylation profiles predicted by DXM are recapitulated in relevant sorted cell subpopulations in DLBCL samples. As proof-of-concept, we have used DXM to identify subpopulation methylation profiles in DLBCL that may correlate with patient prognosis. Since gene promoters with i-DMRs represent multiple methylation profiles in a sample, it is possible that the number of gene promoters with i-DMRs in a sample serves as a proxy of the subclonal heterogeneity. Based on our reasoning, samples at diagnosis with fewer i-DMRs might represent a more clonal disease, which could be more aggressive and more likely to relapse. Since relapse samples go through many changes, including a bottleneck where many of the subclones in the diagnosis sample are lost due to treatment and subsequent expansion of new subclones, as expected there was no clear relationship between the number of i-DMRs at diagnosis and at relapse. Interestingly, a previous analysis of methylation heterogeneity in this cohort found that samples at diagnosis with more epipolymorphisms, regions where epialleles of four or more adjacent CpG sites exhibit high entropy, are more likely to relapse (5). However, since epialleles describe a different intrasample methylation phenomenon than i-DMRs, it is not surprising that samples at diagnosis with fewer i-DMRs are more likely to relapse. While our initial findings based on 18 patients need to be validated in a larger cohort, they illustrate how DXM could be applied to analyze subpopulation methylation profiles in cancer samples.

In summary, here we have developed a new method DXM to accurately deconvolve methylation profiles of allelic subpopulations in genomic-bisulfite sequencing data. We demonstrated its superior performance relative to similar methods, and through several case studies showed how it can be used to study loss of imprinting, X-inactivation and methylation heterogeneity in cancer. We expect that DXM will have high applicability and utility for the analysis of subpopulation methylation in any heterogeneous sample and in the future a similar approach could be adapted to the analysis of cell-free DNA.

## DATA AVAILABILITY

DXM is available under the GNU GPL License on GitHub at https://github.com/edwardslab-wustl/dxm or as a Docker image at https://hub.docker.com/repository/docker/edwardslab/dxm. DXM was written in Python3 and was tested on CentOS7, though it should work on any *nix system. For DLBCL samples, DXM on average used 300Mb memory and processed a sample in less than two hours, using one CPU.

All bisulfite sequencing data has been made available as a GEO accession GSE130556.

## Supplementary Material

gkab516_Supplemental_Files

## Notes

Present address: Jacob R. Gardner, Computer and Information Science, University of Pennsylvania, PA, USA.
