# Supplementary material for: Determining subpopulation methylation profiles from bisulfite sequencing data of heterogeneous samples using DXM
Source: Nucleic Acids Res. 2021 Jun 22;49(16):e93. doi: 10.1093/nar/gkab516 (PMC8450090; doi:10.1093/nar/gkab516)
Supplement: gkab516_Supplemental_Files [file gkab516_supplemental_files.zip › Supplementary_Figures_and_Tables.pdf]

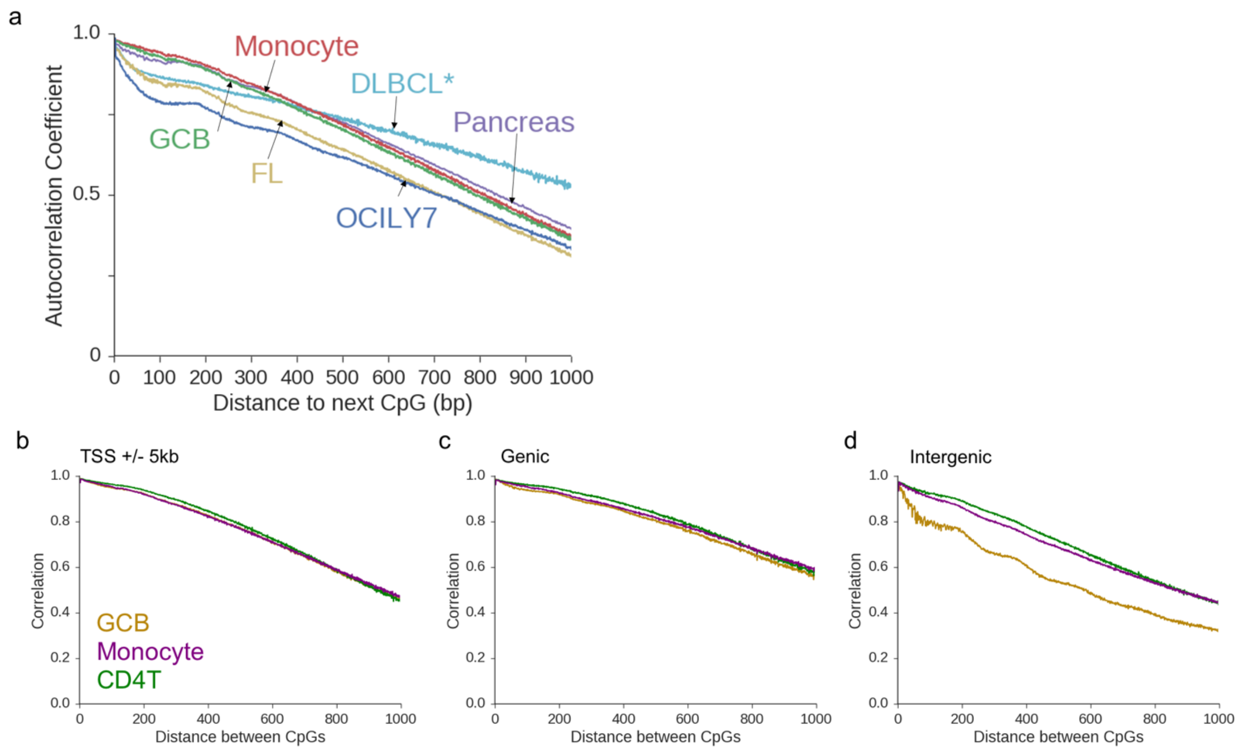

Supplemental Figure 1. Methylation exhibits high degrees of correlation between CpGs in different genomic contexts. **(a)** Correlations of DNA methylation for all CpGs genome-wide. Representative samples were selected for Follicular Lymphoma (FL) and DLBCL. *denotes ERRBS, all other samples are WGBS. **(b-d)** Autocorrelations of DNA methylation between CpGs in the region around the **(b)** TSS, **(c)** genic regions, and **(d)** intergenic regions are shown for three sorted cell types. Genic and intergenic regions do not include the region +/- 5kb around the TSS. The differences in autocorrelation between different cell types in different genomic contexts is similar to the range of autocorrelations calculated for different samples genome-wide.

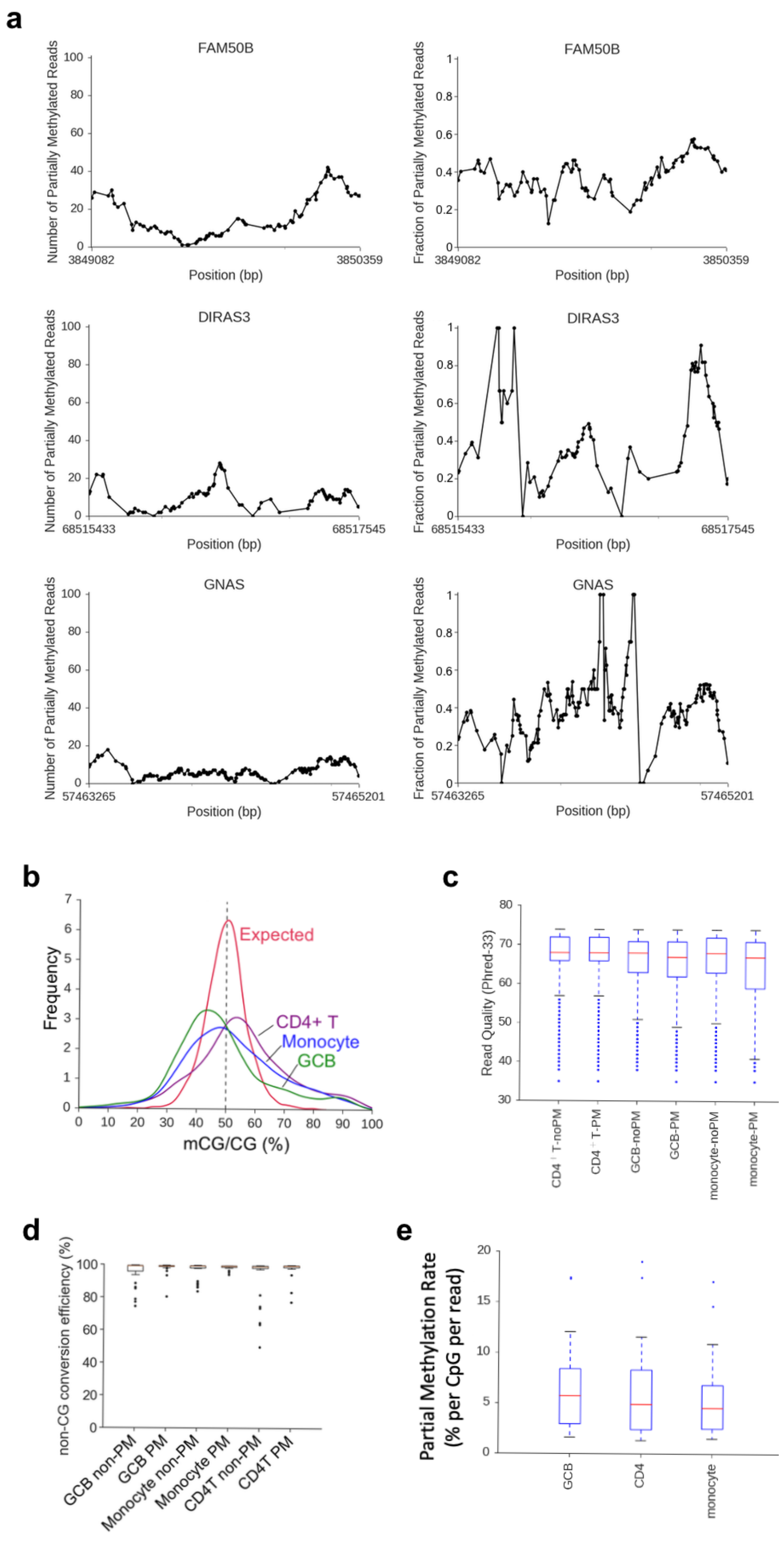

Supplemental Figure 2. Partially methylated reads span imprinted DMRs, cannot be filtered by read quality, and result in wider distributions of detected methylation. a) Number and fraction

of partially methylated reads across the FAM50B, DRAS3, and GNAS imprinted regions in GCB cells. Coordinates denote the start and end of the region. Reads occur throughout the entire locus (ticks every 80bp). **b)** Fractional methylation of CpGs in imprinted DMRs for three sorted cell types. In sorted cell types the distribution is wider than expected for 50% methylation (red dotted line) where the sampling error is modelled using a binomial distribution. All reads encompassing at least one CpG in an imprinted region were considered for analysis. Reads are equivalent to fragments since data are from single-end reads. **c)** Distribution of read quality for partially methylated reads (PM) or those that are fully methylated or unmethylated (e.g. not partially methylated, noPM). The read quality is not substantially different between PM and noPM reads for $CD4^{+}$T cells (d=0.11), GCB cells (d=0.187), and monocytes (d=0.22). d = Cohen's effect size. **d)** Non-CG bisulfite conversion rates for PM and noPM reads. Rates are similar between the PM and noPM groups across three different samples. Reads overlapping the imprinted region were classified as partially methylated (PM) if in the imprinted region, there was at least one CpG that was methylated and one CpG that was unmethylated. If all CpGs shared the same methylation state (all methylated or all unmethylated) in the imprinted region, they were classified as non-partially methylated (non-PM). Next, for each group, conversion efficiency of each imprinted region was computed as the fraction of all non-CG in the region that were methylated, akin to number of non-CG per read * number of reads in the region, though each read can have a different number of non-CG detected. **e)** Average rate (per CpG, per read) of partial methylation in imprinted loci (GCB-8.3%, monocyte-7.7%, $CD4^{+}$T-6.9%). These rates are much higher than the errors due to bisulfite conversion and sequencing.

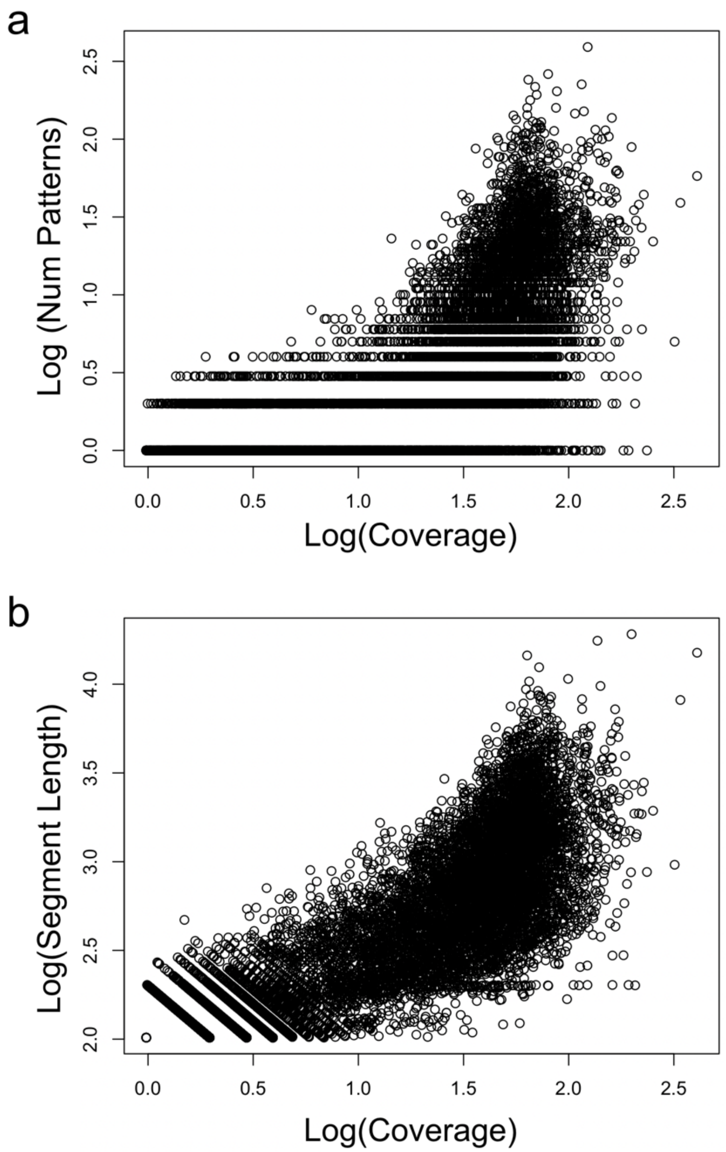

Supplemental Figure 3. MethylFlow analysis of a simulated mixture of HMEC and HCC1954 cells from chromosome 10. (a) The log of the number of patterns identified in the segment (r=0.75, Pearson) and **(b)** the log of the length of the segment (r=0.89, Pearson) are highly correlated with the log of the segment coverage. MethylFlow identifies all possible patterns of a segment, then connects them to form a region. By random chance, as total coverage increases, there may be more possibilities to consider.

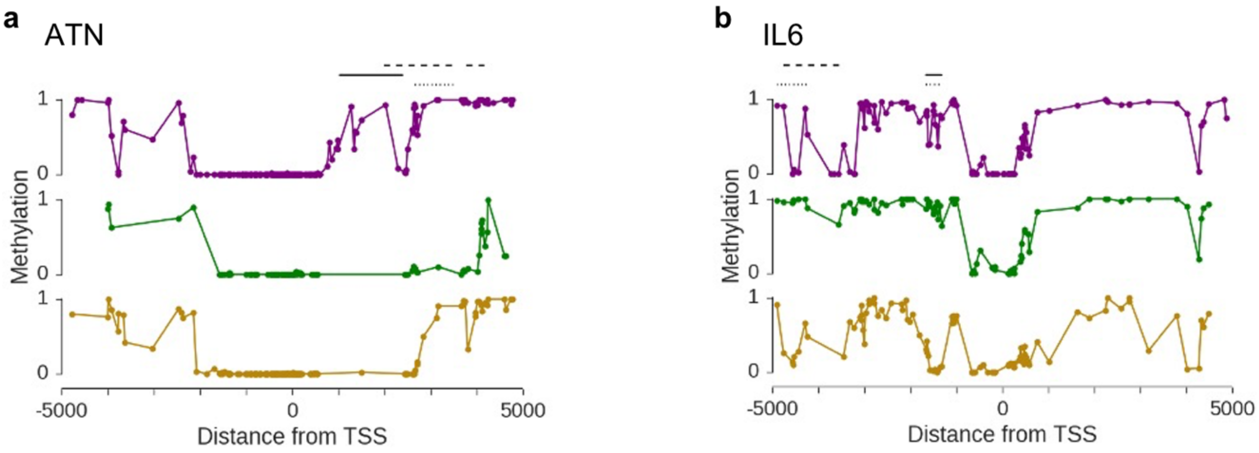

Supplemental Figure 4. Example promoter regions with DMRs that distinguish three cell-types. Methylation of promoter window around the genes ATM **(a)** and IL6 **(b)** are plotted for GCB cells (gold), CD4+ T-cells (green) and monocytes (purple). DMRs are shown as black lines between CD4T-mono (dashed), GCB-mono (solid), and GCB-CD4T (dotted). Provided there is a DMR between each pair of cell-types, the methylation patterns collectively can be considered to distinguish the cell-types. Notably, DMRs can overlap between different pairs of cell-types (e.g. -1664:-1325 for IL6). Promoter windows (+/-5 kb around the TSS) were considered to have distinct methylation patterns if there was a DMR identified between each pair of cells considered. For example, for cell types A, B, and C, a region is considered to have three distinct patterns if there is a DMR between A-B, B-C, and A-C in the region.

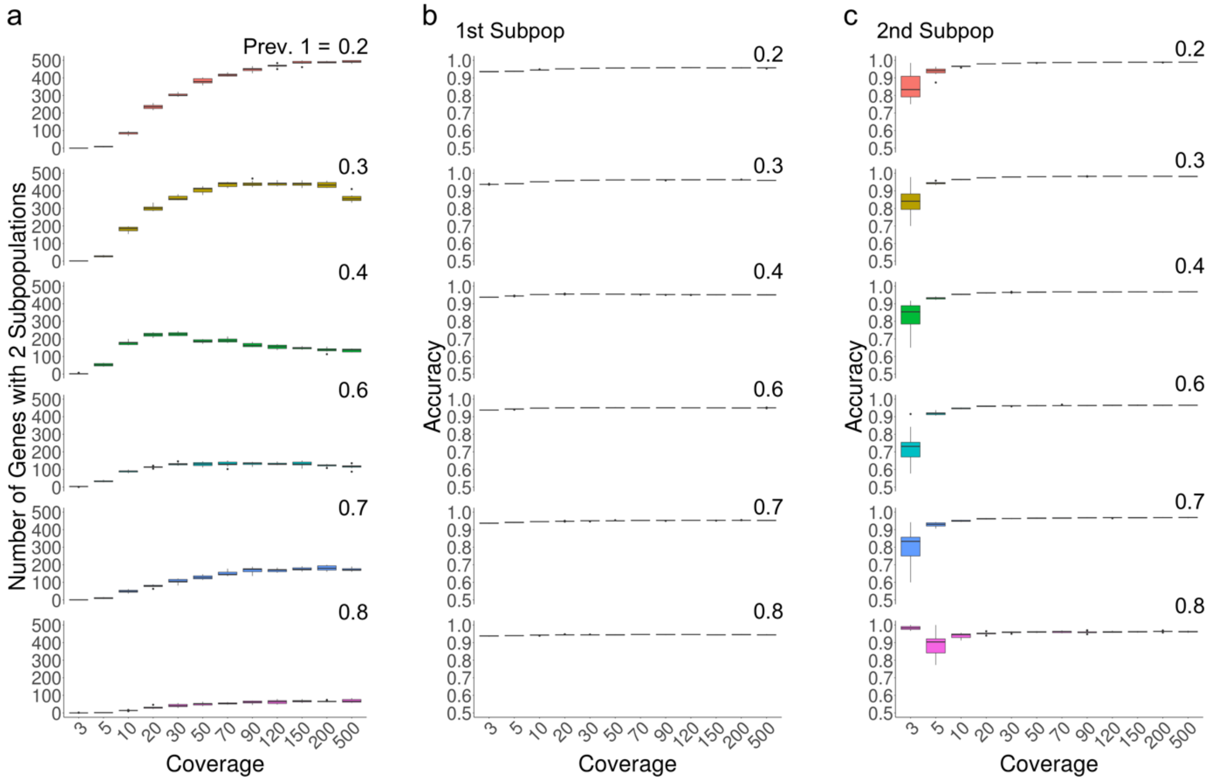

Supplemental Figure 5. DXM performs well on simulated data. DXM was used to deconvolve 720 simulated mixtures of HMEC and CD4+T cells. Methylation values were binarized prior to simulation. The prevalence of HMEC cells in the mixture (Prev. 1) was varied from 0.2 to 0.8. The coverage was fixed for each simulation, and mixed methylation values were modelled based on binomial sampling. For each coverage and prevalence, 10 simulations of 1000 genes were performed. The total number of genes with two subpopulations is shown (**a**) along with the accuracy for the 1st and 2nd subpopulations (**b** and **c**). DXM only separates out a second subpopulation when the accuracy is high.

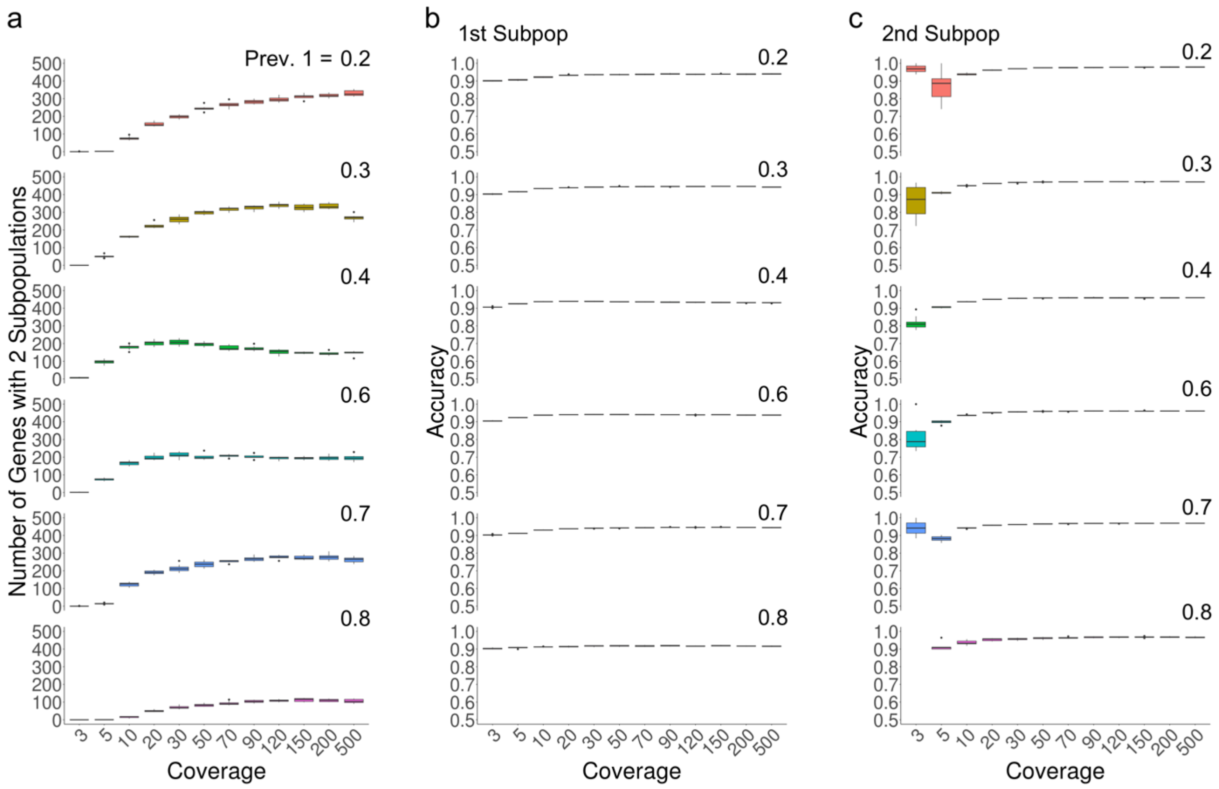

Supplemental Figure 6. DXM performs well on simulated data (dataset mixture 2). DXM was used to deconvolve 720 simulated mixtures of HMEC and HCC1954 cells. Methylation values were binarized prior to simulation. The prevalence of HMEC cells in the mixture (Prev. 1) was varied from 0.2 to 0.8. The coverage was fixed for each simulation, and mixed methylation values were modelled based on binomial sampling. For each coverage and prevalence, 10 simulations of 1000 genes were performed. The total number of genes with two subpopulations is shown (**a**) along with the accuracy for the 1st and 2nd subpopulations (**b** and **c**). DXM only separates out a second subpopulation when the accuracy is high.

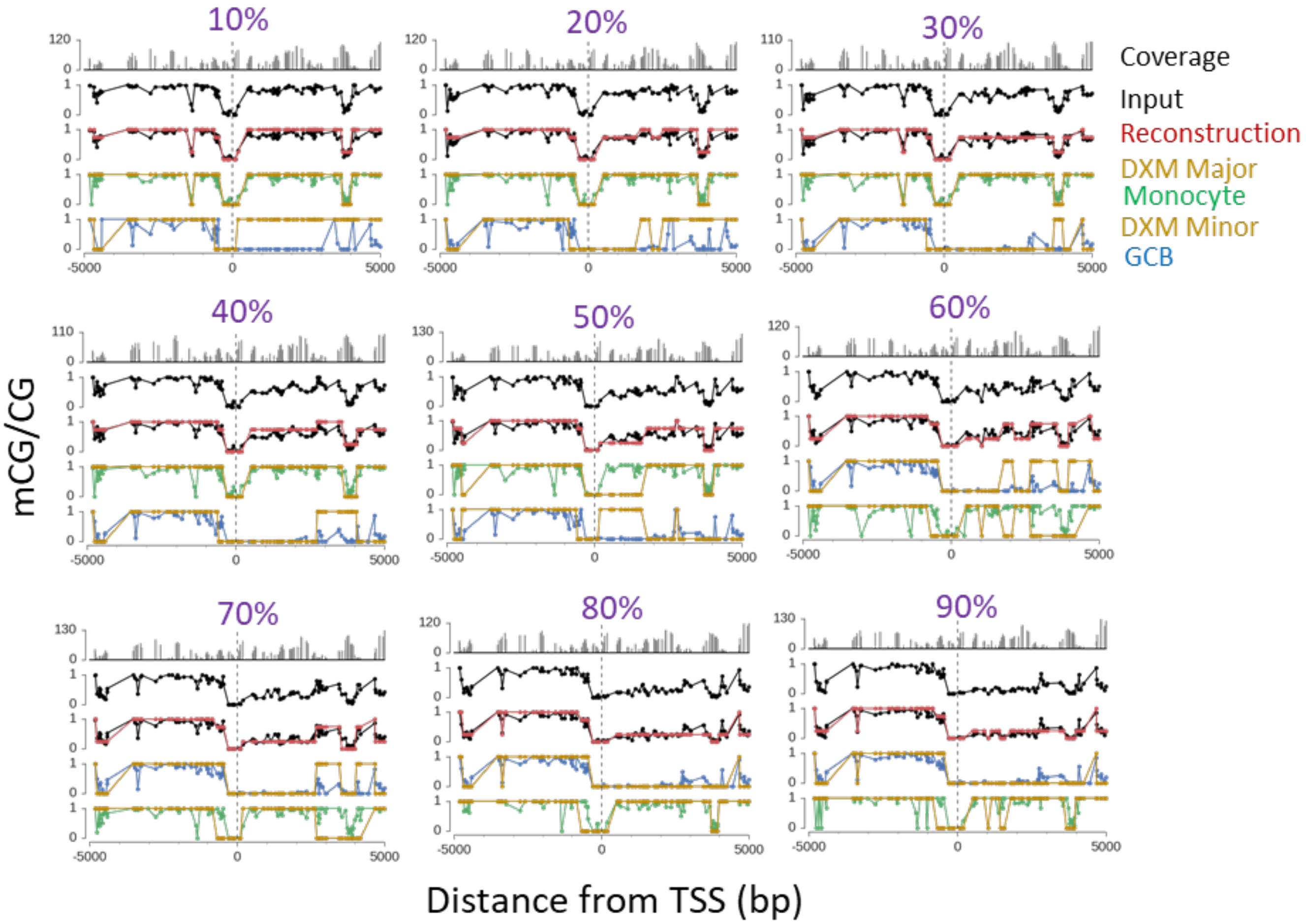

Supplemental Figure 7. DXM performance is high when one subpopulation is greater than 10% prevalence and when both subpopulations are not at the same prevalence (50%). DXM solutions for the CD22 gene across GCB:monocyte mixtures with 55x average coverage and varying prevalence. Percentage denotes GCB prevalence.

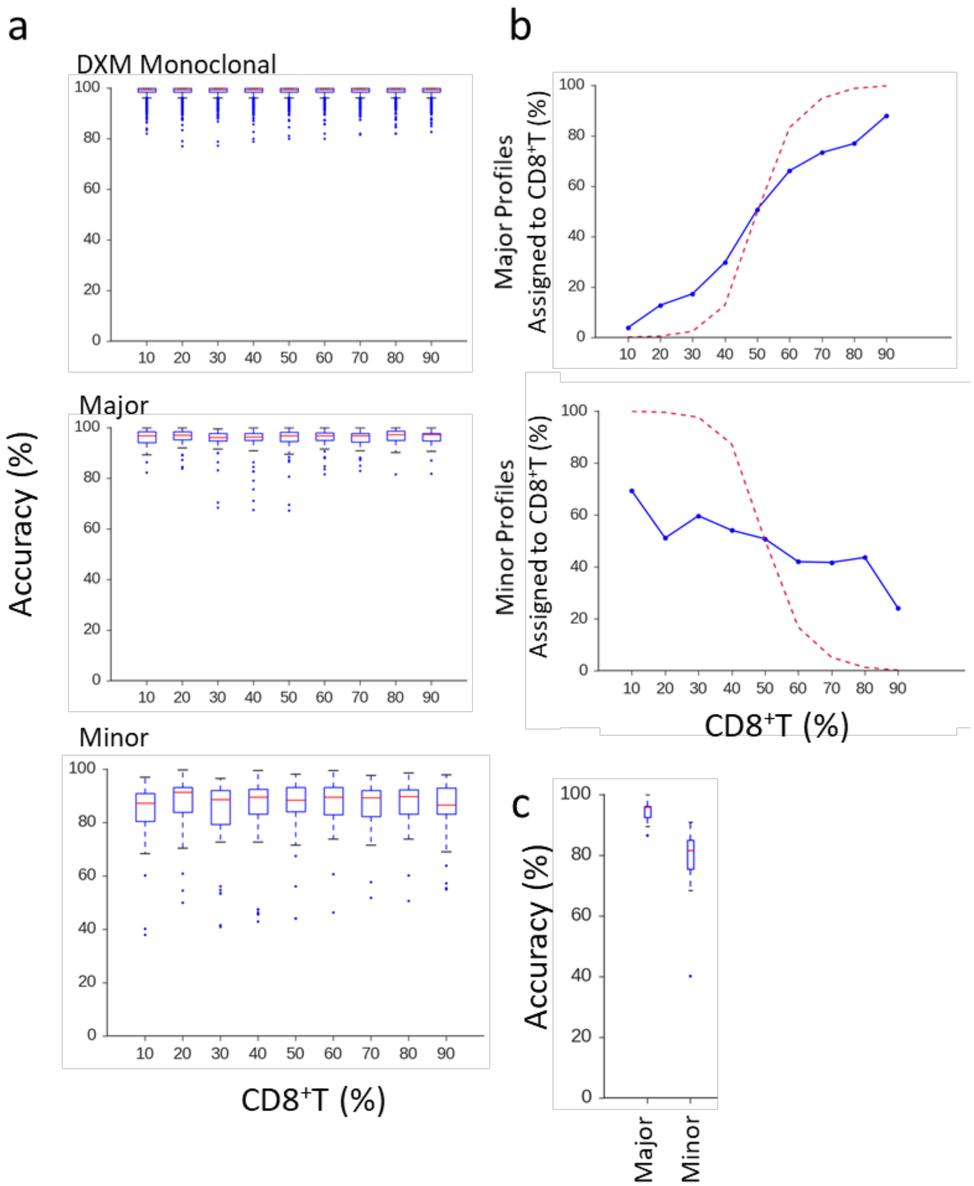

Supplemental Figure 8. DXM accurately deconvolves major and minor subpopulation profiles from 30x mixtures of CD4$^+$T:CD8$^+$T. a) Accuracy of methylation profiles to reference cell types for DXM monoclonal genes, or for the major and minor profiles. **b)** Assignment of major (top) and minor (bottom) profiles to cell-types. CD4$^+$T and CD8$^+$T cells are closer in lineage (435 promoters with DMR) than GCB:monocytes (2,988 promoters with DMR), leading to more difficult assignment. **c)** Accuracy for cases where there both major and minor profiles solved by DXM are assigned to the same cell type (see example in Supplemental Figure 10a)

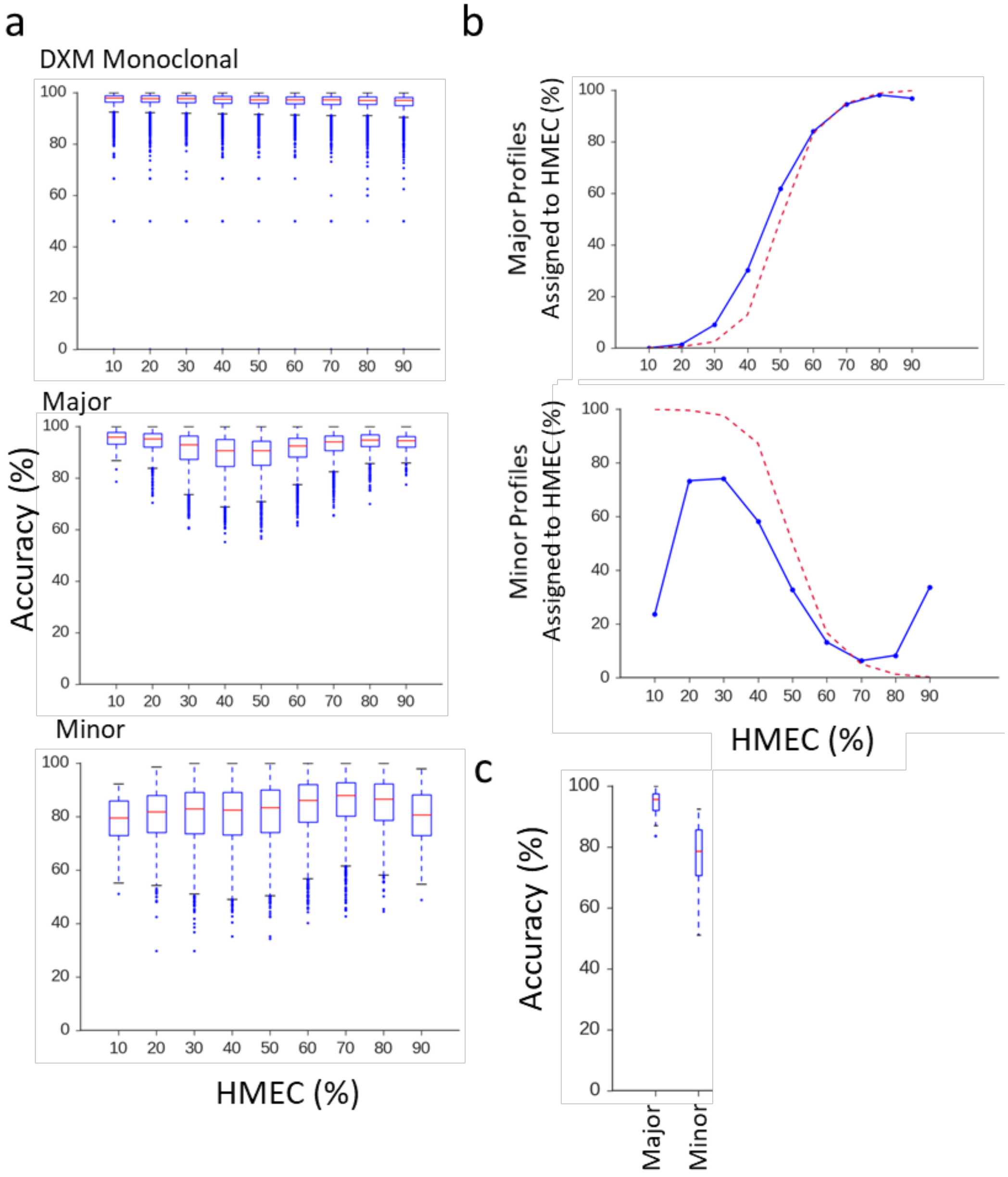

Supplemental Figure 9. DXM accurately deconvolves major and minor subpopulation profiles from 20x mixtures of HMEC:HCC1954. a) Accuracy of methylation profiles to reference cell types for genes with a single profile, or the major and minor methylation profile. **b)** Assignment of major (top) and minor (bottom) profiles to cell-types. **c)** Accuracy for cases where there both major and minor profiles solved by DXM are assigned to the same cell type (see example in Supplemental Figure 10a*)*.

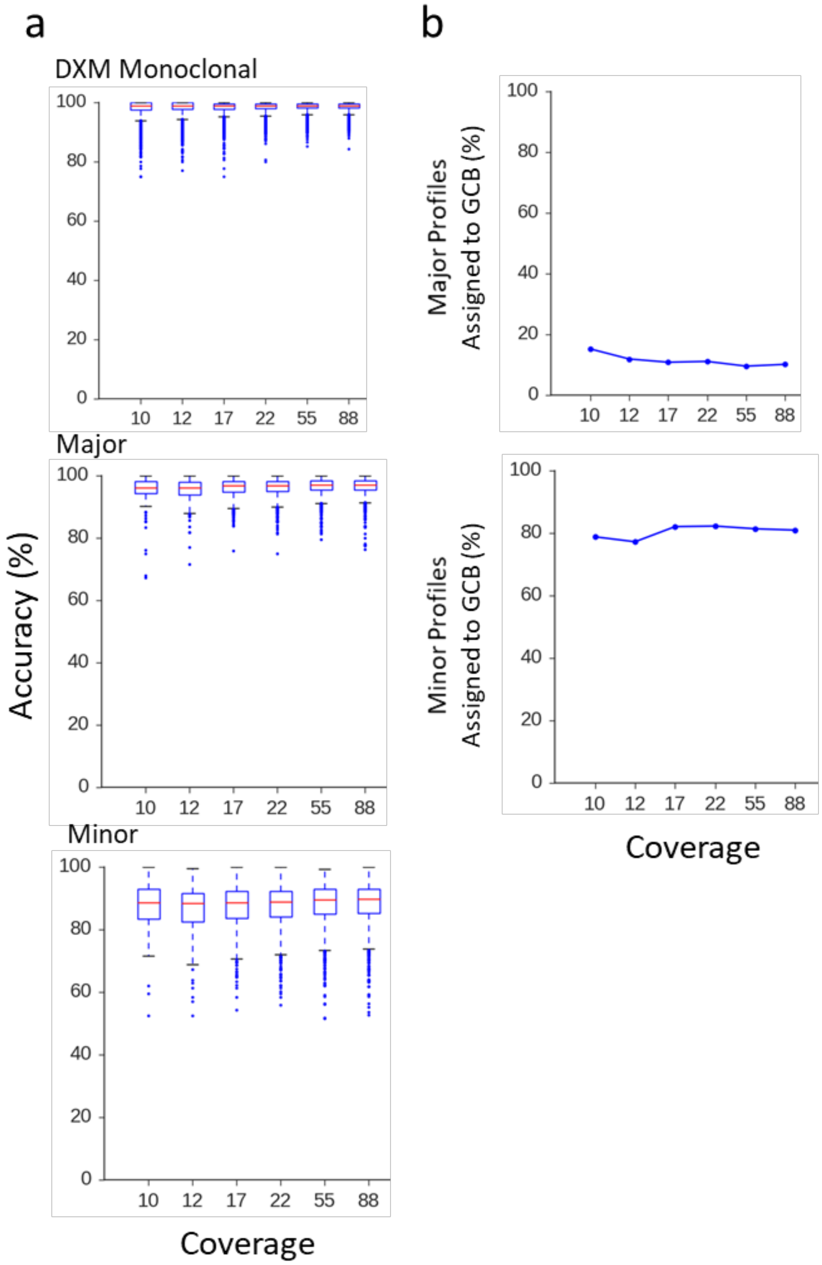

Supplemental Figure 10. Sequencing coverage from 10x-80x does not affect accuracy of reconstructed profiles or assignment of profiles to cell-types. DXM i-DMRs were defined from a GCB-monocyte mixture with fixed prevalence (35:65) and varying average global coverage. **a)** DXM accuracy for genes with one profile (single), or the major and minor profiles. **b)** Percent of all major (top) or minor (bottom) profiles assigned to GCB for simulated mixtures.

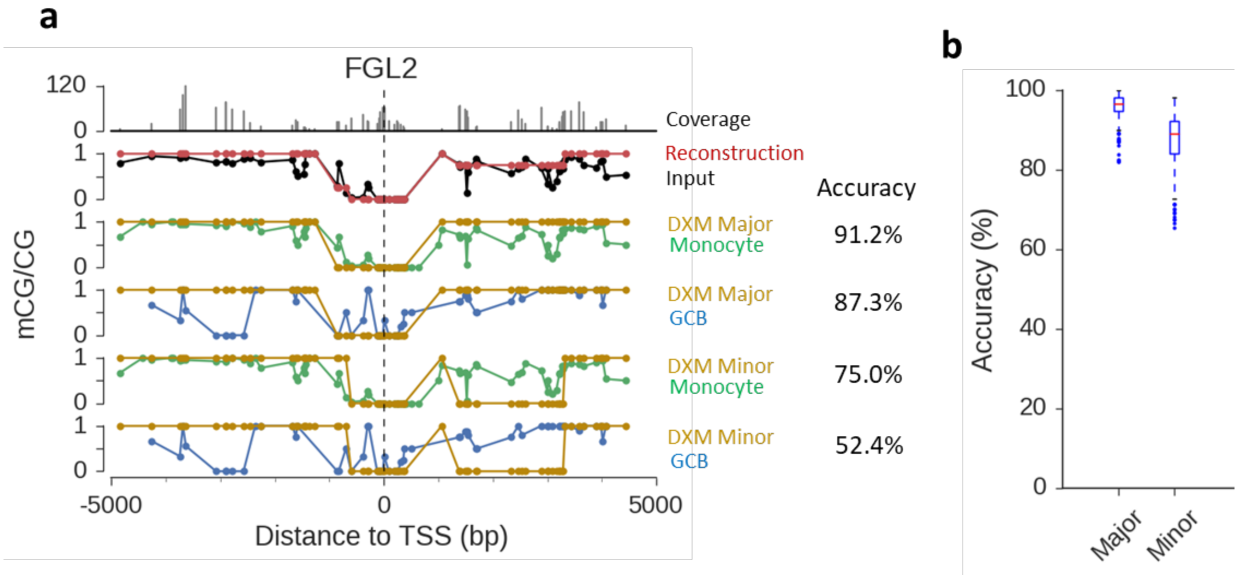

Supplemental Figure 11. DXM solutions for genes with difficult cell-type assignment. a) In a 10:90 simulated mixture of GCB:monocytes, DXM identifies two profiles at FGL2, both of which are closest to reference monocytes. The accuracy of each profile to each potential reference is shown on the right. **b)** Accuracy of DXM major and minor profiles for genes where both profiles share the same closest reference in a 10:90 mixture of GCB:monocytes.

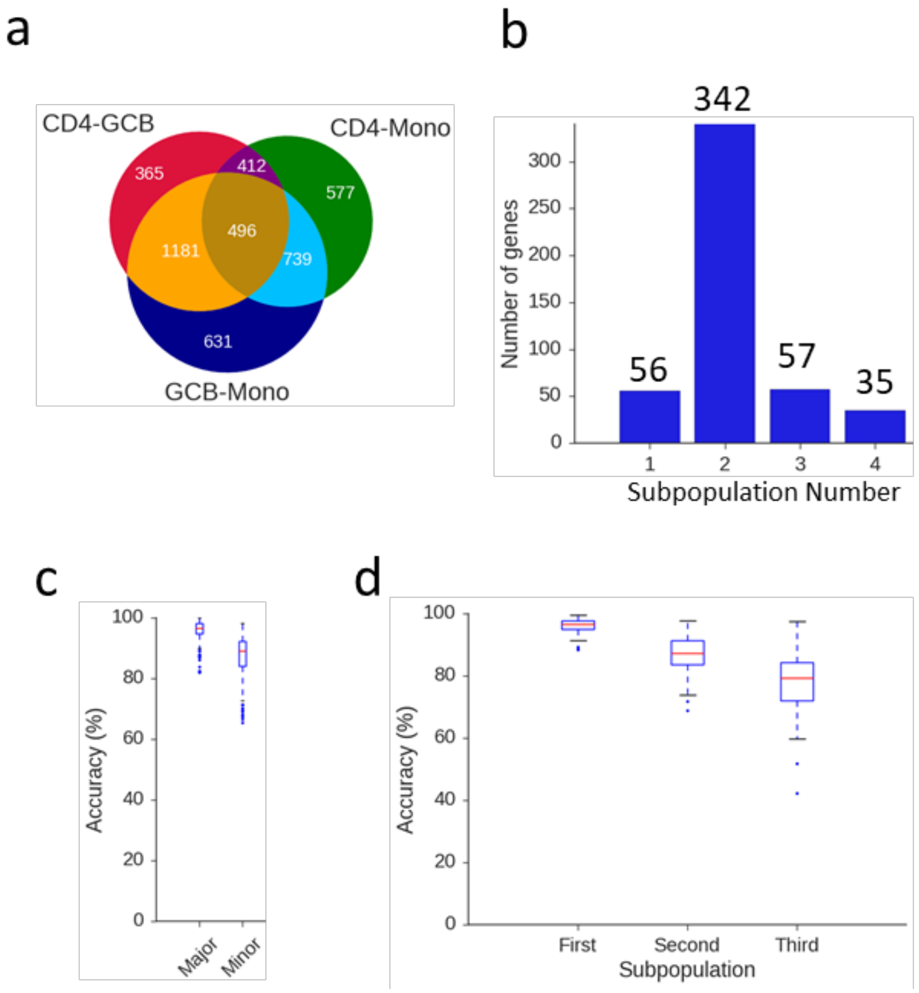

Supplemental Figure 12. Characterization of DXM performance for more than two subpopulations. a) Venn Diagram for genes with a DMR between at least two cell-types (GCB, CD4T, monocytes). Only 496 genes have a DMR between each pair of cell-types (three expected methylation profiles). **b)** Number of methylation profiles identified by DXM for 490 genes with three expected methylation profiles in a 55x 10:25:65 mixture of CD4T:GCB:monocytes. 6 were not present following subsampling used to generate the mixture. **c)** DXM has high accuracy for major (95.9%) and minor (87.4%) profiles at 342 genes where only two methylation profiles were detected. Performance remains high **d)** DXM has high accuracy of first (96.0%), second (87.0%), and third (77.6%) methylation profiles, ordered by descending prevalence, for the 57 genes where DXM found three distinct profiles.

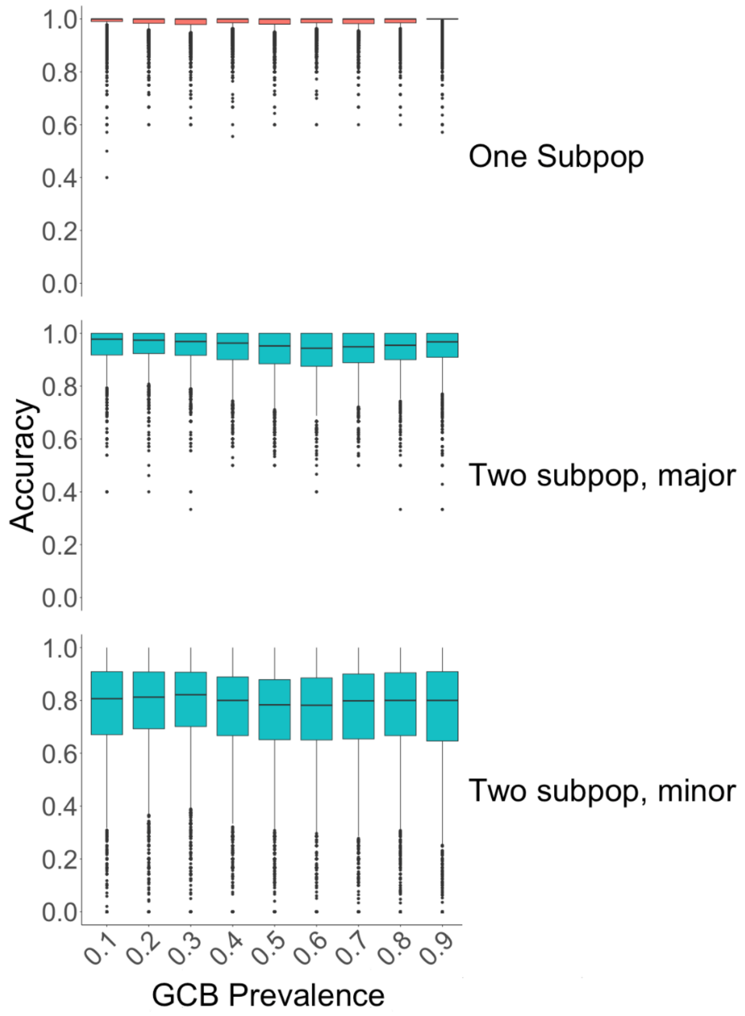

Supplemental Figure 13. DXM accurately deconvolves methylation profiles at enhancers. DXM was used to deconvolve mixtures of GCBs and monocytes. Enhancers were determined as described in the methods. Only enhancers with greater than 20x coverage and at least 5 CpGs are shown.

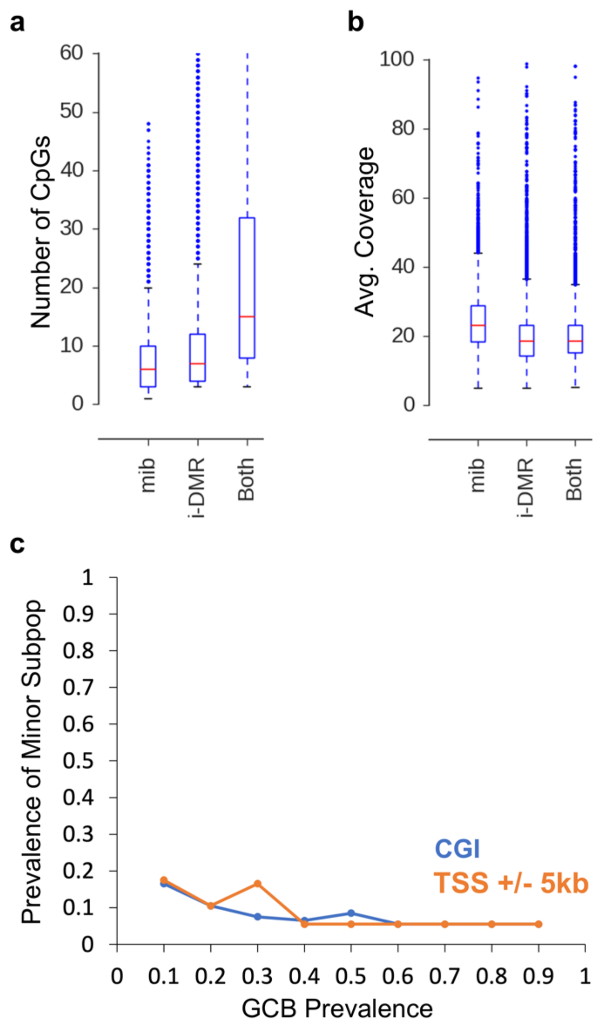

Supplemental Figure 14. Comparison of MP and DXM results. The **(a)** number of CpGs and **(b)** average coverage is shown for the most informative bins (mib) identified only by methylPurify, i-DMRs identified only by DXM, or regions identified by both methods (i.e. overlapping i-DMRs and mib) in a simulated mixture of HMEC and HCC1954 cells. mib identified only by methylPurify tend to have fewer CpGs than i-DMRs identified by DXM or by mib overlapping i-DMRs (Both). This is true even though the coverage is similar. This indicates that regions with strong support are identified similarly by both methods. By definition, mibs must be 300bp, whereas DXM i-DMRs can vary in size. For both number of CpGs and coverage, all groups differ from each other ($p < 0.001$, ANOVA with Tukey's posthoc test). There is a substantial effect size (Cohen's d >0.8) for all comparisons except between "Both" and "i-DMR" for the average coverage (d=0.269). **(c)** Minor subpopulation cell prevalences predicted by MethylPurify for different simulated mixtures of GCBs and monocytes (40x coverage).

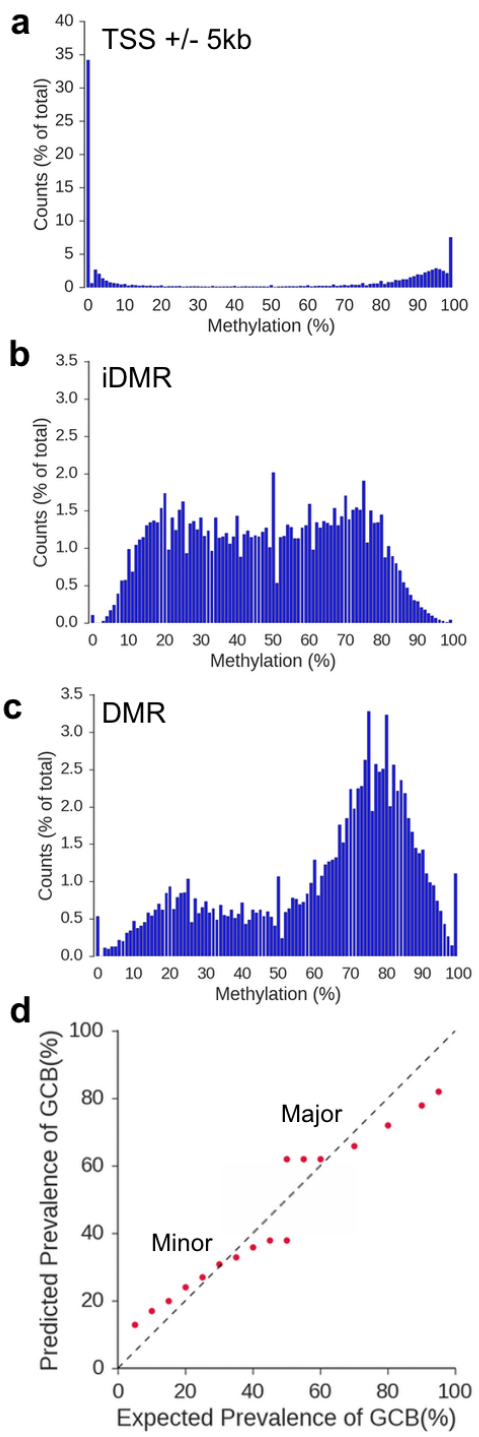

Supplementary Figure 15. DXM accurately measures the cell prevalence. a-c) Methylation distributions of (**a**) the TSS ± 5kb window, (**b**) all iDMRs, and (**c**) all DMRs from a 55x coverage simulated mixture of monocytes and GCBs (20:80). Expected peaks at ~20% and ~80% percent methylation, which can be used to infer a 20% subpopulation, partially appear using iDMRs and fully appear using DMRs. **d)** Estimated prevalences of GCB cells in GCB:monocyte mixtures (55x coverage) using a GCB-monocyte gene signature. Dashed line indicates a perfect estimate.

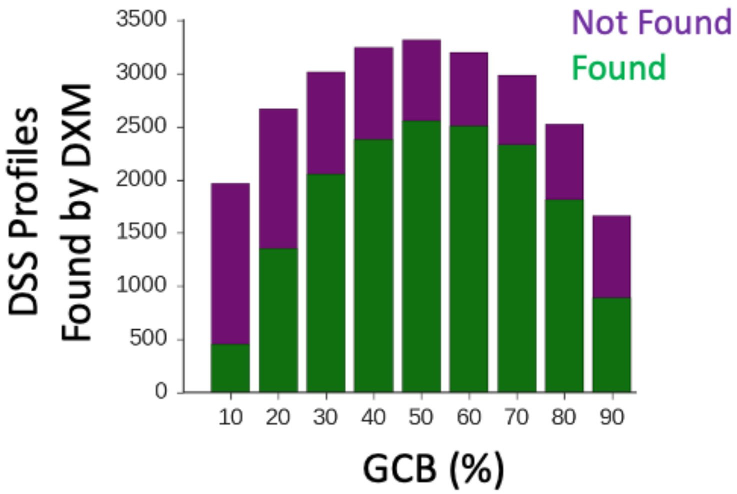

Supplemental Figure 16. DXM i-DMRs recapitulate DMRs identified by DSS. Number of DSS-identified DMRs that are not found (purple) or found (green) by DXM as i-DMRs in subsampled mixtures (35:65 GCB:monocyte). DXM identifies an average of 63.8% of DMRs.

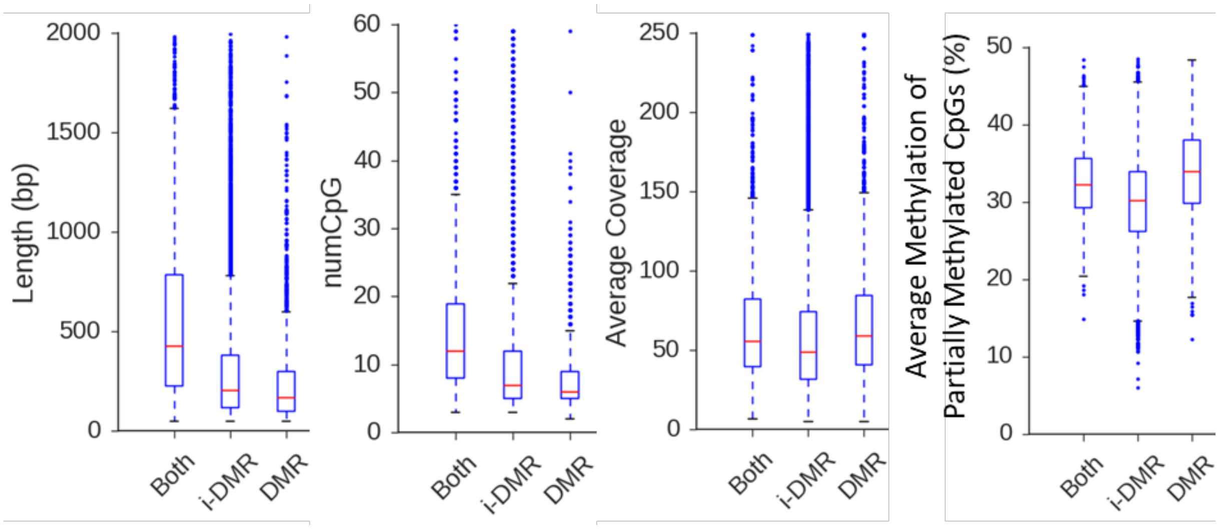

Supplemental Figure 17. DXM i-DMRs and DSS DMRs that overlap are longer and tend to have more CpGs. In a 30:70 simulated mixture of GCB:monocytes, regions identified by both DXM and DSS (Both) tend to be shorter and involve fewer CpG than those identified by DXM but not DSS (i-DMR) or by DSS but not DXM (DMR). To avoid confusion with merging, we considered "Both" as a characteristic of an i-DMR (identified by DXM) that intersects with a DMR (identified by DSS). For length, both vs i-DMR ($p<0.001$ ANOVA with Tukey's posthoc, Cohen's $d=0.81$) and both vs DMR ($p<0.001$, $d=0.79$). For number of CpG, both vs i-DMR ($p<0.001$, $d=0.39$), both vs DMR ($p<0.001$, $d=0.79$). There is no difference in average coverage between Both and DMR, and there is a small difference between Both and i-DMR ($p<0.001$, $d=0.16$) and between Both and DMR ($p<0.001, d=0.21$) for average coverage. For average methylation at partially methylated reads, there is a larger difference between Both and i-DMR ($p<0.001$, $d=0.44$) and between Both and DMR ($p<0.001$, $d=0.66$). There is a small difference between i-DMR and DMR for average methylation ($p<0.001$, $d=0.25$).

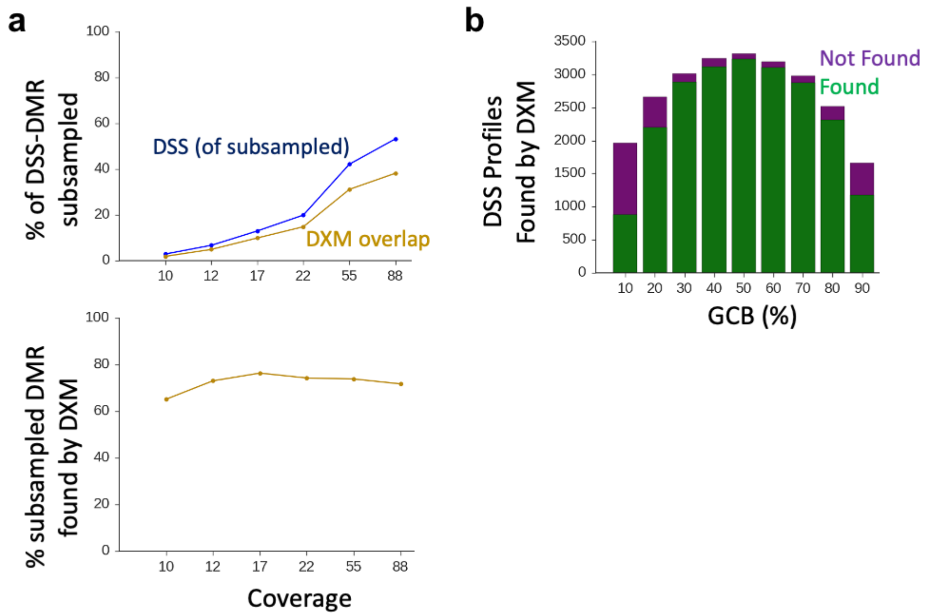

Supplemental Figure 18. DXM i-DMRs recapitulate DMRs identified by DSS. a) Number of total DMRs and DMRs that intersect i-DMR identified in subsampled mixtures (35:65 GCB:monocyte) relative to the number found between reference cell types. As coverage increases, the numbers of both DMRs and i-DMRs increase, but the relative number of DMRs recapitulated by DXM does not. **b)** Number of DSS-identified DMRs that are not found (purple) or found (green) by DXM with a more specific domain (target region). Given specific DMR-locations to consider, DXM identifies an average of 85.9% of DMRs.

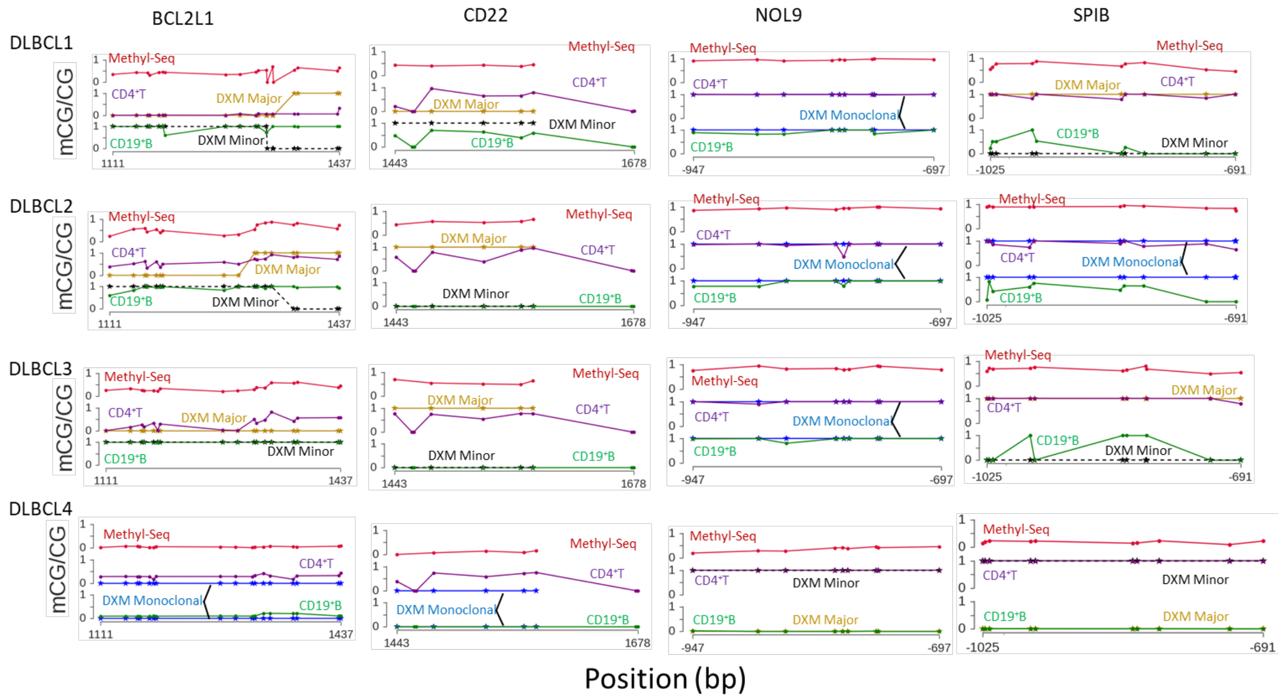

Supplemental Figure 19. Targeted bisulfite sequencing for four genes in sorted CD4$^+$T and CD19$^+$B cells from four DLBCL samples. Each gene had at least one sample predicted to have an i-DMR and at least one without. Methyl-Seq input data (red), DXM predictions (monoclonal blue, major profile gold, minor profile black dash), sorted CD4+ T-cells (purple), sorted CD19+ B-cells (green).

Supplementary Table 1. DLBCL sample characteristics.

DLBCL	Treatment	Diagnosis	Clinical Flow	IHC	FISH
1	Diagnosis	DLBCL-recurrent, nonGCB	REGION A small cells: CD3: 63%; CD19: 37%; Region B large cells: CD3: 37%, CD19: 68%	CD20+, PAX5+, MUM-1+, BCL6 weak in a subset; CD10-	BCL2, MYC, BCL6-
2	Relapse	DLBCL-GCB	LN: CD3 24%; CD19 76%; Tonsil: CD19: 49%; CD3: 51%	CD10+, BCL6+, CD5+, BCL2+, CD20+	None
3	Diagnosis	DLBCL-GCB	Region A low SS: CD3: 82%, CD19: 15%; Region B high SS:CD19 7%	CD20+, CD10+, PAX5+, BCL2+, BCL6 WEAK	None
4	Diagnosis	DLBCL-GCB	CD3: 13%, CD19: 77%	CD10+, CD20+	Igh-BCL2 +, loss of one copy of 8q24

Supplementary Table 2. Primer information for targeted bisulfite sequencing. All coordinates are hg19.

Gene	Chr	Strand	Start	End	Primer-1	Primer-2
CD22	19	+	35821466	35821822	AAAAATGTATAGAGTTGGTTAAATAAAA	CATAAACAAATACCCAACAACCTTTA
SPIB	19	+	50921140	50921537	GGATTTGGAAGATTAGGAGTAGTT	AATCCCCAAAATCATCACCA
BCL2L1	20	-	30309417	30309813	AGGTTAAAGAAAAGGGATATATAAGGG	ACCACAACAACAATTTAAATACC
NOL9	1	-	6615330	6615720	GGGGTTTGAAGTTAGGAAGTATT	AAAAACAAAAACAAAATCCTATTTA

Supplementary Table 3. Cell lines and tissues used for deconvolution of CGIs on the X chromosome. Shown is the fraction of CGIs identified by DXM to have two subpopulations. Data is from the Roadmap Epigenomics Project.

Type	ID	Cell line/Tissue	Sex	Fraction with 2 subpops
Cell Line	E003	H1 ES Cells	M	27.3%
	E004	H1 BMP4 derived Mesendoderm	M	0.0%
	E005	H1 BMP4 derived Trophoblast	M	11.2%
	E006	H1 derived Mesenchymal	M	3.1%
	E007	H1 derived nueronal proginetor	M	12.5%
	E008	H9 Cells	F	84.5%
	E011	hESC derived CD184+ Endoderm	M	11.2%
	E012	hESC derived CD56+ Ectoderm	M	17.2%
	E013	hESC derived CD56+Mesoderm	M	14.3%
	E016	HUES64	M	10.2%
	E017	IMR90 fetal lung fibroblasts	F	94.7%
	E021	iPS DF 6.9	M	2.9%
	E022	iPS DF 19.11	M	10.3%
	E024	ES-UCSF4 Cells	F	91.4%
Primary Tissue	E050	Primary hematopoietic stem cells G-CSF-mobilized Female	F	90.8%
	E053	Cortex derived primary cultured neurospheres	F	92.6%
	E054	Ganglion Eminence derived primary cultured neurospheres	F	94.8%
	E058	Penis Foreskin Keratinocyte	M	0.0%
	E065	Aorta	M	7.2%
	E066	liver	U	15.5%
	E070	Brain Germinal Matrix	M	8.2%
	E071	hippocampus middle	M	14.4%
	E079	Esophogaus	M	16.1%
	E094	Gastric	M	7.1%
	E095	Left Ventricle	M	10.2%
	E096	Lung	F	90.8%
	E097	Ovary	F	92.8%
	E098	Pancreas	M	15.4%
	E100	Psoas Muscle	M	9.6%
	E104	Right Atrium	M	0.0%
	E105	Right Ventricle	M	5.9%
	E106	Sigmoid Colon	M	14.3%
	E109	Small Intestine	M	11.2%
	E112	Thymus	M	0.0%
	E113	Spleen	M	12.5%

Supplementary Table 4. Samples used for deconvolution of imprinted DMRs. The ratio is the fraction of DMRs identified by DXM to have two subpopulations (i.e. imprinted). WIBR datasets are obtained through GEO from Theunissen et al., 2016, Cell Stem Cell. Other datasets are from the Roadmap Epigenomics Project (REP) and Blueprint Epigenome project.

Project	ID	Description	Category	Ratio
REP	E003	H1 ES Cells	ES Cells*	0.80
REP	E004	H1 BMP4 derived Mesendoderm	ES Cells*	0.86
REP	E005	H1 BMP4 derived Trophoblast	ES Cells*	0.78
REP	E006	H1 derived Mesenchymal	ES Cells*	0.81
REP	E007	H1 derived nueronal proginetor	ES Cells*	0.81
REP	E008	H9 Cells	ES Cells*	0.78
REP	E011	hESC derived CD184+ Endoderm	ES Cells*	0.75
REP	E012	hESC derived CD56+ Ectoderm	ES Cells*	0.75
REP	E013	hESC derived CD56+Mesoderm	ES Cells*	0.72
REP	E016	HUES64	ES Cells*	0.75
REP	E017	IMR90 fetal lung fibroblasts	ES Cells*	0.97
REP	E021	iPS DF 6.9	ES Cells*	0.75
REP	E022	iPS DF 19.11	ES Cells*	0.83
REP	E024	ES-UCSF4 Cells	ES Cells*	0.91
REP	E050	Primary hematopoietic stem cells G-CSF-mobilized Female	Primary Tissues	1.00
REP	E053	Cortex derived primary cultured neurospheres	Primary Tissues	0.97
REP	E054	Ganglion Eminence derived primary cultured neurospheres	Primary Tissues	1.00
REP	E058	Penis Foreskin Keratinocyte	Primary Tissues	1.00
REP	E065	Aorta	Primary Tissues	1.00
REP	E066	liver	Primary Tissues	0.94
REP	E070	Brain Germinal Matrix	Primary Tissues	1.00
REP	E071	hippocampus middle	Primary Tissues	1.00
REP	E079	Esophogaus	Primary Tissues	1.00
REP	E084	Fetal Intestine Large	Primary Tissues	1.00
REP	E085	Fetal Intestine Small	Primary Tissues	1.00
REP	E094	Gastric	Primary Tissues	1.00
REP	E095	Left Ventricle	Primary Tissues	1.00
REP	E096	Lung	Primary Tissues	1.00
REP	E097	Ovary	Primary Tissues	1.00
REP	E098	Pancreas	Primary Tissues	0.97
REP	E100	Psoas Muscle	Primary Tissues	1.00
REP	E104	Right Atrium	Primary Tissues	1.00
REP	E105	Right Ventricle	Primary Tissues	1.00
REP	E106	Sigmoid Colon	Primary Tissues	1.00
REP	E109	Small Intestine	Primary Tissues	1.00
REP	E112	Thymus	Primary Tissues	0.97
REP	E113	Spleen	Primary Tissues	1.00
Blueprint	EGAD00001002437	Dendritic	Primary Cells	1.00
Blueprint	EGAD00001002403	NKcell	Primary Cells	1.00
Blueprint	EGAD00001002492	Treg	Primary Cells	0.97
Blueprint	EGAD00001002309	eosinophil	Primary Cells	1.00
Blueprint	EGAD00001002423	erythroblast	Primary Cells	1.00
Blueprint	EGAD00001002311	megakaryocyte	Primary Cells	1.00
Blueprint	EGAD00001002416	memoryB	Primary Cells	1.00
Blueprint	EGAD00001002354	memoryBcs	Primary Cells	1.00
Blueprint	EGAD00001002364	memoryCD4Tcentral	Primary Cells	1.00
Blueprint	EGAD00001002367	memoryCD4Teffector	Primary Cells	1.00
Blueprint	EGAD00001002486	memoryCD8Tcentral	Primary Cells	0.97
Blueprint	EGAD00001002383	memoryCD8Teffector	Primary Cells	0.97
Blueprint	EGAD00001002322	plasmaB	Primary Cells	0.94
Blueprint	EGAD00001002330	precursorB	Primary Cells	1.00
Theunissen et al.	GSM1969070	WIBR3_primed	Primed and Naïve ES Cells	0.59
Theunissen et al.	GSM1969064	WIBR3_4i	Primed and Naïve ES Cells	0.03
Theunissen et al.	GSM1969068	WIBR3_DOX_16	Primed and Naïve ES Cells	0.09

Supplementary Table 5. Ontology analysis with DAVID functional annotation tool for genes with an i-DMR found within 9 sorted cell types (all clusters with at least one element with FDR < 0.05).

Annotation Cluster 1: Enrichment Score 42.57

Category	Term	Count	List Total	Fold Enrichment	FDR
UP_SEQ_FEATURE	domain:Cadherin 6	54	796	16.39829	1.72E-50
UP_SEQ_FEATURE	domain:Cadherin 5	54	796	12.96246	6.74E-43
UP_SEQ_FEATURE	domain:Cadherin 3	54	796	12.1523	5.49E-41
UP_SEQ_FEATURE	domain:Cadherin 4	54	796	12.1523	5.49E-41
UP_SEQ_FEATURE	domain:Cadherin 1	54	796	11.83529	3.20E-40
UP_SEQ_FEATURE	domain:Cadherin 2	54	796	11.83529	3.20E-40
INTERPRO	IPR020894:Cadherin conserved site	54	762	11.63898	4.91E-40
INTERPRO	IPR002126:Cadherin	54	762	11.1458	8.55E-39
INTERPRO	IPR015919:Cadherin-like	54	762	10.96004	2.55E-38
SMART	SM00112:CA	54	458	10.22207	1.31E-37
GOTERM_BP_DIRECT	GO:0007156~homophilic cell adhesion via plasma membrane adhesion molecules	54	711	8.071783	2.35E-30

Annotation Cluster 2: Enrichment Score 18.52

Category	Term	Count	List Total	Fold Enrichment	FDR
UP_SEQ_FEATURE	repeat:PXXP 5	15	796	25.20477	5.20E-16
UP_SEQ_FEATURE	repeat:PXXP 3	15	796	25.20477	5.20E-16
UP_SEQ_FEATURE	repeat:PXXP 4	15	796	25.20477	5.20E-16
UP_SEQ_FEATURE	repeat:PXXP 1	15	796	25.20477	5.20E-16
UP_SEQ_FEATURE	repeat:PXXP 2	15	796	25.20477	5.20E-16

Annotation Cluster 3: Enrichment Score 5.91

Category	Term	Count	List Total	Fold Enrichment	FDR
UP_SEQ_FEATURE	domain:Peptidase S1	19	796	4.393493	4.62E-04
INTERPRO	IPR001314:Peptidase S1A, chymotrypsin-type	19	762	4.095197	0.001224
INTERPRO	IPR001254:Peptidase S1	19	762	3.85631	0.003014
SMART	SM00020:Tryp_SPc	19	458	3.535693	0.007241

Supplementary Table 6. Sequencing results and relevant DXM results.

DLBCL	numCpG	Average Coverage	Non-CpG Conversion (%)	Average Coverage in TSS±5kb window	i-DMR	Genes with i-DMR
1	4.8e6	53.2	98.9	58.7	4,746	6,758
2	4.8e6	53.0	98.9	59	3,785	6,087
3	4.9e6	54.4	98.8	60.5	6,790	9,167
4	4.8e6	56.8	99.0	62.6	4,808	6,896

Supplementary Table 7. Number of i-DMR identified in analysis of 31 DLBCL samples (Pan et al., 2015, Nature Communications)**.**

Sample	i-DMR	Genes with i-DMR
1D	10,113	2,961
2D	9,002	2,885
3D	7,341	2,044
4D	12,907	3,827
5D	14,564	5,182
6D	4,700	1,603
7D	5,300	1,664
8D	15,703	3,899
9D	11,738	3,309
10D	10,276	2,912
11D	6,477	2,269
1R1	12,427	2,654
1R2	7,167	2,952
1R3	14,929	4,041
2R	11,543	3,448
3R	3,506	948
4R	11,387	3,160
5R	15,380	4,744
6R	3,708	1,306
7R	5,218	1,685
8R	18,200	4,005
9R	14,685	3,502
10R	10,771	2,791
11R	10,034	3,386
NR1	13,658	4,182
NR2	12,236	3,850
NR3	14,215	4,368
NR4	12,151	3,768
NR5	9,952	3,111
NR6	14,015	4,186
NR7	12,289	3,493
